# Optical Biosensors for Label-Free Detection of Small Molecules

**DOI:** 10.3390/s18124126

**Published:** 2018-11-24

**Authors:** Riikka Peltomaa, Bettina Glahn-Martínez, Elena Benito-Peña, María C. Moreno-Bondi

**Affiliations:** Departamento de Química Analítica, Facultad de Ciencias Químicas, Universidad Complutense de Madrid, E-28040 Madrid, Spain; rpeltoma@ucm.es (R.P.); ab.glahn@ucm.es (B.G.-M.); elenabp@ucm.es (E.B.-P.)

**Keywords:** label-free, optical biosensor, small molecule, surface plasmon resonance, surface-enhanced Raman spectroscopy, interferometry, evanescent wave, optical fiber

## Abstract

Label-free optical biosensors are an intriguing option for the analyses of many analytes, as they offer several advantages such as high sensitivity, direct and real-time measurement in addition to multiplexing capabilities. However, development of label-free optical biosensors for small molecules can be challenging as most of them are not naturally chromogenic or fluorescent, and in some cases, the sensor response is related to the size of the analyte. To overcome some of the limitations associated with the analysis of biologically, pharmacologically, or environmentally relevant compounds of low molecular weight, recent advances in the field have improved the detection of these analytes using outstanding methodology, instrumentation, recognition elements, or immobilization strategies. In this review, we aim to introduce some of the latest developments in the field of label-free optical biosensors with the focus on applications with novel innovations to overcome the challenges related to small molecule detection. Optical label-free methods with different transduction schemes, including evanescent wave and optical fiber sensors, surface plasmon resonance, surface-enhanced Raman spectroscopy, and interferometry, using various biorecognition elements, such as antibodies, aptamers, enzymes, and bioinspired molecularly imprinted polymers, are reviewed.

## 1. Origin and Occurrence of Small Molecules

Small molecules can be defined as low molecular weight organic molecules which are typically less than 1000 Da in size. This category includes a wide variety of different chemical compounds, of either natural or pharmaceutical origin, many of which are biological, pharmacologically, or environmentally relevant, which makes detection and quantification of these molecules important in many disciplines. Naturally, nearly every cell contains a collection of 100 to 200 different low molecular weight organic molecules, including the common amino acids, nucleotides, sugars, and their phosphorylated derivatives. Additionally, there exists a wide variety of small biomolecules which are specific to certain types of cells or organisms, for example, many plants contain so-called secondary metabolites which include compounds that give plants their characteristic scents, and compounds such as morphine, nicotine, and caffeine that are valued for their physiological effects on humans [1]. On the other hand, small synthetic molecules, man-made or produced by synthetic biology [2], have been extensively applied in a broad variety of fields including pharmaceutical, clinical, environmental, or food analysis, to name a few.

Many small molecules are well-known contaminants in food, feed, and other agricultural products. For example, mycotoxins, which are produced as secondary metabolites by filamentous fungi, include more than 500 different small molecules with different physicochemical properties and various effects ranging from cancer to acute toxicity and developmental defects. Agriculturally, the most critical mycotoxins are aflatoxins, fumonisins, trichothecenes, ochratoxins, and zearalenone family which are common contaminants in crops worldwide [3,4]. Algal toxins, phycotoxins, and cyanotoxins, in turn, are produced by toxicogenic microalgae and cyanobacteria, and they can enter the marine food chain via phytoplankton and subsequently to humans by contaminated seafood. Several small molecule toxins originating from seafood are known to cause severe illnesses, such as paralytic shellfish poisoning, puffer fish poisoning, and neurotoxic shellfish poisoning, and it has been reported that seafood poisonings are increasing in frequency and new intoxications are emerging [5,6]. All the aforementioned toxins are known to cause, besides a threat to human and animal health, but also substantial economic losses in aqua- and agriculture, and many of them fall under national and international regulations.

Along with the naturally produced contaminants, many synthetic low molecular weight compounds are environmentally and pharmacologically significant. For example, biological and chemical warfare agents which can be lethal even at low levels are some of the most feared weapons of mass destruction. Nerve agents, which are usually organophosphates, have rapid and severe effects on human and animal health due to their ability to block acetylcholinesterase (AChE) that is essential for the normal functioning of the nervous systems. Also, many pesticides and insecticides belong to the same chemical class of organophosphates, and they possess the same mode of action as nerve agents but are less hazardous [7]. For example, chlorpyrifos, a broad spectrum pesticide which belongs to the group of organophosphates and is used worldwide in a wide range of crops, presents a major concern for potential environmental contamination and overuse, improper storage or disposal of these molecules [8,9].

Pharmaceuticals, either of natural or synthetic origin, are ubiquitous substances of interest not only in clinical medicine but also in drug screening and environmental safety. Small molecules, in particular, are of great interest in pharmaceutical research, for example, because they can cross the blood-brain barrier. Detection of many drugs is diagnostically and clinically relevant but is also of interest in pharmaceutical sciences for drug screening and development of new biotherapeutics, as well as for studying the activity, kinetics, and stability of pharmaceuticals [10]. Therapeutic drug monitoring, which aims to optimize the pharmacological response of a drug while avoiding adverse effects, measures the drug concentrations in a biological matrix and with appropriate interpretation affects the prescribing procedures [11]. On the other hand, after administration, many drugs are excreted by the patients into wastewater, and in fact, rather often many pharmaceutical products and residues have been found in wastewaters, surface waters and even drinking water [12]. Pharmaceuticals vary from antibiotics to non-steroidal anti-inflammatory drugs, such as ibuprofen, or anti-cancer drugs which are of particular concern not only for human health but also for the environment due to their unique properties in combination with poor biodegradability [13,14]. On the other hand, many small molecules can behave as endocrine disrupting chemicals (EDCs), i.e., they interfere with the body’s endocrine system and produce adverse developmental, reproductive, neurological, and immune effects in both humans and wildlife [15]. According to the World Health Organization (WHO), EDCs and potential EDCs consist mainly of man-made compounds found in various materials, such as pesticides, metals, additives, or contaminants in food and personal care products, and they are a public health issue worldwide [16].

Conventionally, many small molecules are detected by chromatographic methods which usually provide high sensitivity and specificity [9]. However, due to the high cost, bulky instrumentation, and required expertise, these methods are not suitable for every purpose; whereas, biosensors can offer a cheaper and faster alternative. Myriad of different biosensors and bioanalytical assays have been reported for the diverse group of small molecule analytes, ranging from traditional enzyme-linked immunosorbent assays (ELISAs) [17,18] and lateral flow tests [19] to microarrays [20] and fluorescent [21] or electrochemical sensors [22], just to mention a few examples. Some of the advantages of biosensors over classical methods for small molecule detection include real-time monitoring, high specificity, fast response times, reduced consumption of organic solvents and sample manipulation, portability, compactness, and easy operation avoiding the need of skilled personnel [23].

## 2. Biosensors for Small Molecule Detection

Generally, biosensors consist of a bioreceptor, the recognition element responsible for capturing the target analyte, and a transducer, whose properties are altered upon analyte binding [24]. In the context of this review, the majority of the methods can be defined as affinity biosensors since they are based on a specific biorecognition element which is capable of binding to the analyte. Antibodies continue to be one of the most used bioreceptors due to their exceptional specificity and sensitivity, but also nucleic acids, aptamers, peptides, and molecularly imprinted polymers (MIPs) are widely used. On the other hand, catalytic biosensors are based on a bioreceptor, such as an enzyme or whole cell, which is capable of recognizing the analyte and transforming them into a product through a chemical reaction [25]. Usually, the bioreceptor is immobilized on the surface of a transducer where the biorecognition event can be monitored by different transduction schemes which measure the binding event. The binding event can produce, for example, an increase in mass, or a change in the electrical resistivity or refractive index of the surface, which can be monitored by various detection methods, such as mechanical, electrical, or optical signals.

Optical transducers are based on measuring a change in the optical properties in the presence of the analyte, such as absorption, reflectance, emission, or interferometric pattern, which can be recorded by a photodetector. Many optical biosensors require the use of labels, for example, fluorescent dyes, enzymes, or nanoparticles, which are used as a means to measure the biorecognition event. Although such label-based methods are widely used and usually very sensitive, they inevitably require the involvement of a label and a chemical conjugation step to link the label to the biorecognition event. The necessity of labeling makes these methods limited by the success and efficiency of the conjugation step, which furthermore in some cases might even alter the biorecognition event. Thus, label-free biosensors can offer some significant advantages and better accuracy over label-based methods since label-free biosensors do not require the use of a label to monitor the binding event. Advantages of label-free methods include simplicity and speed of the measurement procedure; these methods enable real-time monitoring of the binding reaction, thus giving access to the kinetic and thermodynamic parameters of the molecular recognition process [26,27].

Development of biosensors for small molecule detection represents particular challenges which might not be an issue with larger analytes. Firstly, small molecules are challenging targets for many recognition elements, in particular for antibodies since haptens alone fail to stimulate the immune system responsible for antibody production [28]. Consequently, hapten-specific antibodies are usually selected using the hapten conjugated to a larger carrier molecule, which at times results in antibodies specific for the conjugate rather than for the free hapten. Furthermore, small molecules which are components of physiological pathways, such as amino acid, are not immunogenic. Recombinant antibodies have been presented as an interesting alternative for monoclonal and polyclonal antibodies, and several recombinant antibody-based immunoassays and sensors have been reported during the last two decades [29,30]. Potentially, recombinant antibodies can overcome some of the limitations of their conventional counterparts; they can be selected from naïve repertoires avoiding animal immunization and modified by genetic engineering to improve their binding or include reactive groups or tags intended for conjugation or protein purification. Because of their small size, recombinant antibody fragments can contribute to decreased non-specific binding and lower steric hindrance compared to the intact antibody. However, despite some interesting properties, recombinant antibodies rarely show better or even similar affinities than conventional antibodies [31], which limits their use for the detection of small molecules which are present at low concentrations and thus require recognition elements with high affinity.

Alternatively, biosensors can be based on several other bioreceptors or bioinspired recognition elements. Aptamers are short synthetic DNA or RNA molecules which form a three-dimensional structure which allows them to bind target molecules with high specificity and sensitivity [32]. Aptamers are readily synthesized and modified for immobilization or labeling purposes, and owing to their small size, high chemical and thermal stability, and low price, aptamers can be an intriguing alternative for antibodies. Nonetheless, once again, it can be particularly challenging to find good aptamer binders for analytes with small size [33]. Thus, aptamer-based label-free methods often rely on different signal amplification methods to compensate for the shortcomings of the recognition element [33]. Enzymes which were among the first recognition elements used in biosensors are a rather obvious choice as a recognition element for targets which are known enzyme inhibitors, for example, organophosphates [9,34]; however, their use can be limited by poor stability or loss of activity as a result of the immobilization. Molecularly imprinted polymers (MIPs), also known as plastic antibodies, are artificial materials, and although strictly speaking, they do not match the description of a biosensor which by definition relies on biological recognition element, MIPs are an exciting option for biosensor development due to their high physical and chemical stability, robustness, low cost, and ease of preparation [35]. Molecular imprinting allows the design and preparation of custom-made polymeric materials that contain binding sites with selective affinity to the analyte, similar to some biological receptors, such as antibodies, enzymes, or other protein receptors. For MIP synthesis, the selected print molecule, which may be the analyte or a substitute molecule, interacts via covalent or non-covalent bonds with functional monomers that polymerize in the presence of a crosslinker. Once the template molecule is removed, the resulting three-dimensional supramolecular structure contains specific recognition sites that are complementary to the template molecule or/and target molecule.

**Figure 1:**
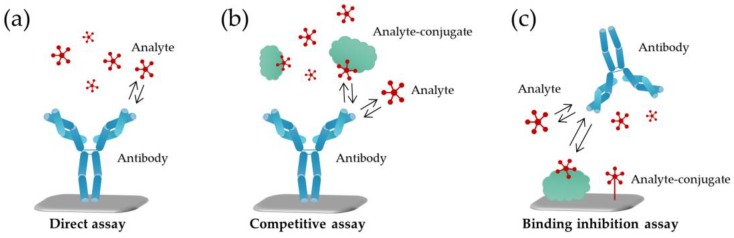
Schematic presentation of assay formats generally used for small-molecule detection. (**a**) In a direct assay, target analyte binds to the recognition element, e.g., the antibody which is immobilized on the sensor surface; (**b**) In a competitive assay, the analyte competes with its conjugate for the binding to the immobilized recognition element; (**c**) In a binding inhibition assay, similarly the analyte and analyte-conjugate compete for the binding, but the analyte-conjugate is the one immobilized on the sensor surface either directly via a linker or as a protein-conjugate. Also, other recognition elements besides antibodies are applied to biosensor development using the same assay formats.

In their simplest format, label-free biosensors are based on direct detection of the target which binds to the recognition element immobilized on the sensor surface (Figure 1a). Compared to small molecules, binding of large molecules usually produces a larger response which is often roughly in proportion to the mass of the molecule. Thus, for direct detection, the requirement is that the analyte of interest produces a sufficient response in a required concentration range, which in practice means that the molecular weight of the target must be large enough to generate a measurable signal change. Generally speaking, the sandwich assay format can be used to improve the response and the assay sensitivity; however, small molecules which are composed of only one epitope are not suitable for this approach [36]. Alternatively, small molecules can be measured indirectly using either a competitive or an inhibition detection format (Figure 1b,c). 

In the competitive format, the sensing surface is coated with the recognition element while the analyte and its conjugated analog compete for a limited number of binding sites on the surface. In the inhibition detection format, reversely, the analyte-conjugate is immobilized on the surface, and the recognition element is added together with the analyte in solution [25]. Regardless of the assay format, or the recognition element of choice, the immobilization step to the sensor surface is critically important to all biosensor configurations. The functionalized biosensor surface should provide high specific binding of the target analyte while maintaining low non-specific binding or cross-reactivity towards interfering molecules present in the same samples.

The repertoire of methods based on optical label-free detection is vast and impressive. In this review, we aim to revise the latest developments in the field with particular attention to solutions that improve the methods for small molecule detection, or present innovative alternatives to overcome some of the limitations mentioned above. Significant research efforts in the field of evanescent wave and fiber optic biosensors, surface plasmon resonance (SPR) and localized surface plasmon resonance (LSPR) biosensors, as well as surface-enhanced Raman spectroscopy (SERS), and interferometry are described.

## 3. Fiber Optic and Evanescent Wave Biosensors

Evanescent wave systems have found broad applicability and flexibility for biosensor design as they confine the interactions between light input/output and fluidics inflow/outflow to a single interface [37]. Evanescent wave biosensors can be based on the use of cylindrical or planar waveguides. Several fiber optic configurations, including tapered optical fibers, photonic crystal fibers, hollow-core fibers, or long-period fiber gratings have been proposed to improve the interaction between the evanescent field and the sensing layer. In most cases, the cladding of the fiber is modified by a sensing layer and any change in the optical or structural properties of the material, such as the refractive index, thickness or absorption will change the transmission properties of the fiber [38]. In absorption sensors, light absorption of the evanescent wave by a sensing layer will result in a decrease of the guided light in the fiber core. In luminescent sensors, the evanescent light will excite the luminescent molecules located near the waveguide surface, and the emitted light will be captured and guided by the waveguide. Therefore, the background signal from the bulk sample can be minimized, and these methods are usually very sensitive and selective for the detection of small molecules in comparison with label-free techniques [37,39,40]. However, only a few molecules can be detected directly using this approach without the use of labels.

Optical waveguide light mode spectroscopy (OWLS) is an optosensing technique that applies the evanescent field for the in situ and label-free study of surface processes at the molecular level [41]. In this approach, polarized laser light is diffracted by a grating and incoupled into a thin waveguide. The incoupling resonance takes place at precise angles, depending on the characteristic optical parameters of the sensor chips and the refractive index of the external medium. Photodiodes detect the intensity of the incoupled light. The effective refractive index is determined from the resonance incoupling angle which is detected with high accuracy and allows the evaluation of the layer thickness and coverage (or mass) of the adsorbed or bound material with excellent sensitivity. OWLS has been applied for example to the analysis of mycotoxins ochratoxin A (OTA) and aflatoxin B_1_ (AFB_1_) in grain samples using direct and indirect assay formats. Higher sensitivities were obtained using the inhibition assay with an immobilized antigen, and the response range was between 0.5 and 10 ng/mL for both mycotoxins. The same conclusion was obtained for the analysis of trifluralin and zearalenone, two environmental endocrine disrupters, vitellogenin, an endocrine marker [42] in environmental samples and deoxynivalenol (DON), a mycotoxin in wheat [43]. The competitive immunosensor was shown to be more sensitive for the target compounds than the noncompetitive ones. This behavior can be explained considering that the OWLS signal is sensitive to relative masses bound to the waveguide surface. Binding of an antibody to an immobilized antigen conjugate produces a higher signal than binding of small analytes, as in the case of the mycotoxins or the herbicide, to an immobilized antibody. The same group applied the OWLS immunosensor to the analysis of AFB_1_ in 60 spice paprika samples from different countries, finding that 16 samples were contaminated with AFB_1_, and 9 samples contained AFB_1_ above the official maximum residue level (5 μg/kg) set by the European Commission. Excellent correlation was observed with the results obtained by ELISA and high-performance liquid chromatography (HPLC) with fluorescent detection [44].

**Figure 2:**
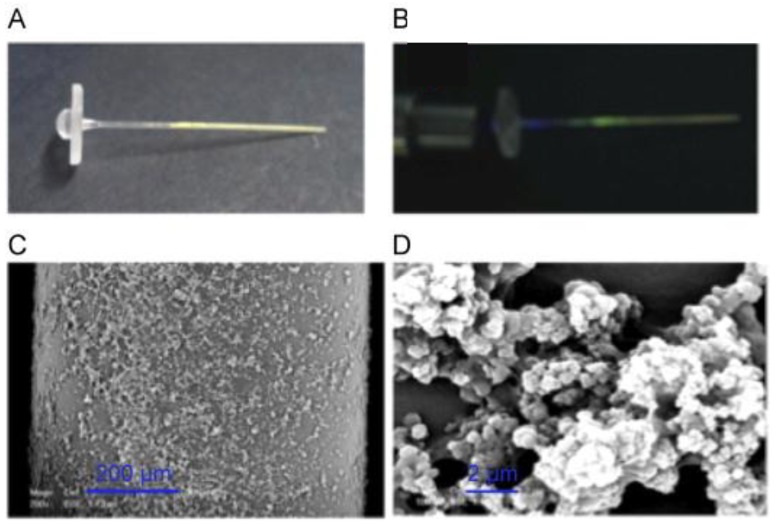
(**A**) Photograph of the polystyrene evanescent wave fiber optic waveguide coated with fluorescent 2,4-D MIP particles using polyvinyl alcohol as glue. (**B**) Injection of the light with a fiber optic bundle and collection through the lens (λexc = 410 nm; λem = 515 nm). (**C**,**D**) SEM micrographs of the surface of the 2,4-D-MIP-coated optical fiber (reprinted from [45] with permission from Elsevier).

In a completely different approach, the Haupt group [45] reported the development of disposable evanescent wave fiber optic sensors by coating 4-cm long injection-molded tapered polystyrene waveguides with 2,4-dichlorophenoxyacetic acid (2,4-D) (Figure 2) or citrinin selective MIPs containing a fluorescent signaling monomer, *N*-(2-(6-4-methylpiperazin-1-yl)-1,3-dioxo-1*H*-benzo[*de*]isoquinolin-2(3*H*)-yl-ethyl)acrylamide, which can be excited by the evanescent wave. Polymer coating on the cylindrical waveguide was carried out either by in situ photopolymerization of the MIP on the fiber using the evanescent wave, or by dip coating with the MIP particles. An increase in fluorescence intensity proportional to the analyte concentration was observed in the presence of the target compound with a limit of quantification of 1 nM for 2,4-D and good selectivity to the herbicide. The biomimetic sensors obtained by in situ polymerization of the MIP were less sensitive than those obtained using the MIP particles.

Barrios et al. [46] demonstrated the applicability of aluminum nanohole arrays (NHAs), deposited onto a microscope coverslip, for sensing applications. The pre-polymerization mixture was deposited onto the NHAs, and the light was efficiently confined inside favoring the photopolymerization of submicron-sized MIP patterns selective to rhodamine 123 (R123), as a model template molecule. The final size could be tuned by changing the dose of green radiation applied during the polymerization step. Evaluation of the selective recognition of R123 by the imprinted polymer was carried out by fluorescence lifetime imaging microscopy with single photon timing measurements. A similar approach has been applied to the development of biosensors for the detection of biotin as a model analyte, in combination with glass, polycarbonate compact discs (PC CDs) or transferable aluminum NHAs onto flexible pressure-sensitive adhesive tapes using label-free SPR measurements [46,47].

Margheri et al. [48] described the detection of heavy metal ions by self-assembling a monolayer of a fluorescent indicator which was quenched in the presence of Hg(II) ions on the external surface of an optical fiber waveguide formed by a metal (Au)-dielectric (SiO_2_) bilayer known as metal–clad optical waveguide (MCOW). The thin Au metal layer (~20 nm) guided the zero-order TE and TM modes with the lowest possible losses, and the thickness of the dielectric layer was selected to propagate the same modes. On the other hand, the fluorescent layer and the metal were separated by the SiO_2_ layer, thus avoiding the metal-induced fluorescence quenching. The authors estimated a limit of detection of 150 nM and response time of approximately 2 min which were in the same order of magnitude as the best ones reported in the literature for this metal ion. Further improvements were proposed by the substitution of Au with an Ag layer and increasing the number of sensing sites by enriching a hydrogel volumetric matrix.

Genetically modified organisms have also been coupled to optical fibers for the development of label-free biosensors for the sensitive and selective detection of a wide range of compounds of interest in different fields such as food analysis, environmental monitoring, and clinical diagnostics, among others [49,50,51,52,53]. Sensor performance depends on the introduction of a reporter gene into the host cell, whose expression is modulated by the interaction of the analyte with the molecular recognition element, and promoter sequences [54]. Widely used reporter genes include *lux* (bacterial luciferase), *Luc* (firefly luciferase), GFP (green fluorescent protein), and *lacZ* (β-galactosidase). The activity of the reporter protein can be monitored upon the addition of the corresponding (e.g., bioluminescent, fluorescent) substrate or directly as in the case of the GFP [55].

In a different approach, non-genetically modified microorganisms, such as green microalgae based on mutant algae clones which are the result of evolutionary strategies, have also been applied to the development of biosensors for the analysis of pesticides or heavy metals. Two different approaches have been used for this purpose. The first was based on monitoring the variation in the algal chlorophyll fluorescence at 682 nm, as a result of the inhibition of the algal photosystem II by the pollutant [56,57]. Alternatively, herbicide concentration was evaluated by monitoring the inhibition of the photosynthetic O_2_ production by the target compound using an oxygen optode [58].

In addition, several fiber optic sensors are based on SPR which requires the use of a metal-dielectric interface. Different optical structures have been reported for SPR-based fiber optic biosensors, including D-shaped, de-cladded, end mirror or tapered fiber structures [38]. Some relevant examples of fiber optic SPR sensors and the basis of SPR sensing are described in the following section.

## 4. Surface Plasmon Resonance

Surface plasmon resonance (SPR) is one of the most advanced and most used label-free detection techniques because of the high sensitivity and versatility of the method, as well as the possibility for a real-time read-out and direct measurement of binding kinetics. As a result of tremendous research on SPR during the last decades and the availability of advanced commercial instruments, SPR-based biosensors can be considered as a landmark label-free biosensing platform for characterizing and quantifying biomolecular interactions [36,59]. SPR sensors measure changes in the refractive index which occur at the surface of a metal film where electromagnetic waves, called surface plasmons, propagate upon illumination. Since surface plasmons are highly dependent on the geometry of the plasmonic structure and the environmental parameters, changes such as biomolecule binding to the surface will change the plasmon mode [60]. Prism couplers are most commonly used, but alternatively, optical excitation can be based on waveguide couplers, diffraction gratings, or integrated optical fibers [61]. Prism-based coupling requires bulky instrumentation and thus is not compatible with portable platforms or point-of-care devices. Instead, optical fibers are low cost and ideal for miniaturization, which has made them as the subject of intense research [62,63]. A major contribution to SPR-based biosensors is the use of metal nanostructures which are smaller than the incident wavelength and when interacting with light waves generate a resonance phenomenon known as localized surface plasmon resonance (LSPR). One of the most interesting features of LSPR is the possibility of tuning the SPR intensity by varying the size, shape, composition, and environment of the nanostructures which can be for example metal nanoparticles, carbon nanotubes or nanowires [61].

Direct detection of small molecules by SPR is challenging due to their small size and subsequently the small change in the refractive index produced by analyte binding, which generally leads to measurements with too low signal-to-noise ratios [64]. Nevertheless, direct detection is more straightforward and has several advantages over indirect methods, such as shorter assay times, lower sample volumes, no need for the conjugated analyte competitor, and the possibility for direct kinetic measurements [65]. Advances in SPR instrumentation, such as detection systems with lower noise and improved fluidics as well as advanced immobilization methods and new sensor chips, have simultaneously decreased the total noise and improved the signal intensities and reproducibility [64]. The most advanced commercial SPR instruments, such as Biacore, claim the detection of analytes down to 100 Da [66] and although the early immunosensors for small molecule detection relied almost inevitably on the competitive assay formats, during the last decade several examples of direct small molecule detection have been reported.

The performance of SPR sensors is highly dependent on the chemical interface and bio-functionalization, as well as the optical, electrical and structural features of the instrument [47]. The choice of a recognition element indisputably has a direct effect on the assay sensitivity and specificity, but moreover, the immobilization of the interacting molecule, either the recognition element or the analyte-conjugate, is crucial. A robust biomolecule coating on the sensor surface should be stable and, in many cases, preferably regenerable. It is critical to ensure that the surface coating and the immobilization do not degrade the biological activity of the recognition element or result in steric hindrance, and a sufficient number of molecules must be immobilized to ensure reliable signals simultaneously minimizing the non-specific binding to the surface [50]. The following sections discuss SPR-based methods reported for small molecule detection using different recognition elements, and Table 1 summarizes some of the most interesting approaches from recent years.

### 4.1. Antibody-Based SPR Sensors

Owing to their unique properties and an immense variety of possible specificities antibodies continue to be the most used recognition element in SPR sensors, although alternative options for small molecule detection, mostly aptamers or molecularly imprinted polymers (MIPs), have also been reported [36]. Despite the extensive use of both SPR and antibodies, only a few examples of direct SPR-based immunosensors have been described, and direct SPR methods for small molecule detection still might suffer from poor sensitivity. For example, Tomassetti et al. [67] compared a direct-flow SPR immunosensor for ampicillin detection with a competitive amperometric immunosensor which showed better sensitivity and wider dynamic range than direct detection by SPR. SPR sensor was more selective, as well as faster and simpler in the analysis, compared to the amperometric method in the concentration range from 10^–6^ M to 10^–2^ M. In comparison, an LSPR sensor composed of core-shell nanosensors for the detection of atrazine was prepared using polystyrene nanospheres, as the “core” of the nanochip, and a gold layer which was thermally deposited onto the core as the “shell”. After studying the response of three types of nanosensors with different Au film and shell dimensions, the authors biofunctionalized one of the nanosensors with an atrazine specific antibody and proved direct detection of atrazine at 10 ng/mL [68].

**Figure 3:**
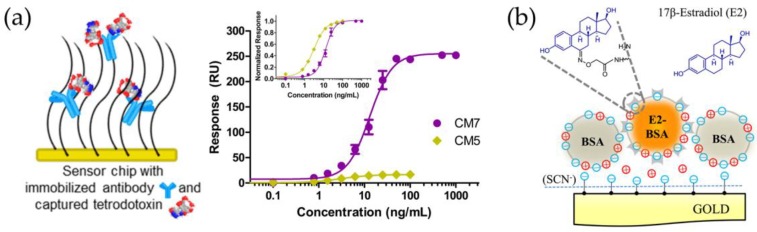
(**a**) For tetrodotoxin detection, the antibody was covalently immobilized onto carboxymethylated (CM) dextran surface (left). A comparison of high-density CM7 and low-density CM5 surfaces for antibody immobilization (right). (**b**) Schematic representation of the immobilization of estradiol(E2)-BSA conjugate within the BSA matrix utilizing the electrostatic levitation phenomenon for forming a “fluid”-like interfacial membrane with rotation freedom and in-plane mobility of membrane components ((**a**), reprinted from [69], copyright (2014) American Chemical Society; (**b**), reprinted from [86] with permission from Elsevier).

The importance of the recognition element immobilization procedure has been established in several applications. For example, direct detection of tetrodotoxin was performed with Biacore T200 instrument using CM5 and CM7 sensor chips for antibody immobilization. Introduction of higher conjugation substrate, CM7, provided significantly greater response compared to CM5 substrate with a lower number of antibody binding sites, although analysis with CM5 gave better sensitivity than CM7 (EC_50_-values 2.98 ng/mL and 12.45 ng/mL, respectively) (Figure 3a). Compared with the inhibition assay, the direct detection method provided not only faster and simpler analysis, but also four times the lower limit of detection (LOD 0.091 ng/mL for direct assay on CM5 chip, LOD 0.38 ng/mL for inhibition assay) [69]. In a different approach, an SPR immunosensor for ochratoxin A (OTA) was constructed on a nano-size gold hollow ball with a dendritic surface that was used to immobilize anti-OTA monoclonal antibody. OTA detection was reported in the range of 0.05–7.5 ng/mL with a detection limit of 0.01 ng/mL. According to the authors, the three-dimensional network of the gold hallow microspheres provided more space for the protein adsorption and thus improved the sensitivity compared to the SPR sensor with gold nanoparticles [70]. Direct SPR sensor for benzoylecgonine (BZE), a major cocaine metabolite, consisted of high-affinity monoclonal antibody immobilized with high density to a sensor chip which contained a polycarboxylated hydrogel as a three-dimensional immobilization matrix. Detection of BZE in oral fluid could be performed within 180 s with BZE concentrations as low as 4 μg/L in filtered oral fluid-buffer (1:4) samples [65].

Nonetheless, the majority of antibody-based SPR sensors are based on an inhibition assay format where a target-conjugate is immobilized onto the sensor surface. Although direct assays can be considered more ideal than the competitive inhibition assays, it should be noted that the latter can avoid some of the drawbacks related to antibody immobilization, such as potential changes in the native structure, improper orientation of the immobilized antibody, or a deteriorated affinity, all of which have direct effects on the assay performance. Therefore, immobilization of the target, either directly or conjugated to a carrier molecule, might provide a more robust sensor surface [71]. A variety of different surfaces chemistries have been used with different binding site densities, but probably the most used and the most versatile surface is the Biacore CM5 sensor chip which consists of carboxymethylated dextran covalently attached to a gold surface and can be used to covalently couple molecules via amine, thiol, aldehyde, or carboxyl groups [66]. During the last 10 years, a wide range of SPR immunosensors for small molecules have been reported using the inhibition assay, and several of them have presented sub-regulatory detection limits including validation with sample analysis. A specially designed multi-microchannel SPR sensor for the detection of herbicide 2,4-D was based on an array of thin Au-films, and a multi-microchannel plate with a flow-cell and the protein-conjugate was immobilized merely by physical adsorption onto the sensing surface [72]. Majority of reported methods are based on protein-conjugated target which is covalently attached to the sensor surface. For example, contamination of mycotoxins nivalenol (NIV) and deoxynivalenol (DON) in wheat was tested using a sensor chip with DON–bovine serum albumin (BSA)-conjugate immobilized using a conventional amine coupling method. The competitive inhibition assay with a monoclonal antibody that cross-reacts with NIV and DON provided IC_50_ values of 28.8 and 14.9 ng/mL for NIV and DON, respectively [73]. Likewise, SPR immunosensors with BSA-conjugated domoic acid (DA) and cortisol were used to analyze DA contamination in clam extracts [74] or cortisol levels in saliva [75], respectively, whereas others have reported sensors with ovalbumin (OVA)-conjugated enrofloxacin (ENRO) [76] as well as patulin [77] and benzylpenicillin [78] conjugated to glutamine-binding protein for target immobilization. Alternatively, protein-conjugated small molecules have been immobilized on a sensor surface using self-assembled monolayers (SAMs) to obtain good stability high degree of reproducibility. SPR immunosensors using SAMs or mixed SAMs to immobilized protein-conjugated targets have been reported at least for the detection of atrazine [79], ractopamine [80] and antibiotics amikacin [71] and fluoroquinolones [81]. Robustness of the SAMs has been proven as the surface could be reused at least 40 times over span of three days [81] or even up to 150 times [80]. In the work of Herranz et al. [82], several assay formats with different immobilization strategies were evaluated for the detection of microcystin-LR (MCLR). Compared to the protein-conjugated or biotinylated target, direct immobilization of MCLR onto an amine-SAM-functionalized chip was concluded to provide the best performance. Alternatively, detection of saxitoxin [83,84] and HT-2 toxin [85] has been accomplished by directly immobilizing the target to the biosensor chip via amino-coupling. Recently, a self-tuning interfacial architecture for estradiol detection was described with a decreased detection limit and widened dynamic range [86]. The novel immobilization strategy was based on a “charged” surface where a flexible and structurally variable architecture was created by a variety of weak electrostatic interactions utilizing the electrostatic levitation phenomenon (Figure 3b).

Also, a few SPR biosensors using recombinant antibodies have been reported for small molecules, mainly mycotoxins and other biotoxins with sub-regulatory detection limits, but most SPR-based methods in the literature in combination with recombinant antibodies consists of examples where SPR is used as a method to characterize novel antibodies and determine the binding affinities [87,88,89,90,91,92,93,94]. For these applications, the target is immobilized directly [87], via a biotin-linker [88], or as a protein-conjugate [89,90,91,92,93,94] to the sensor chip. It has been noted that the structural format of recombinant antibody fragments, as well as chip functionalization, strongly affects the SPR response. Townsend et al. [95] concluded that direct coating and the Fab antibody format would be optimal since multimerization of scFvs might result in incomparable SPR sensorgrams. Although recombinant antibodies still often suffer from lower affinity than monoclonal antibodies, which consequently limits the sensitivity levels that can be reached, they offer some intriguing characteristics, including their small size which could provide immobilization at high density, and the possibility to genetically engineer specific tags or reactive groups to facilitate the immobilization [31,96,97].

Finally, numerous examples have demonstrated that nanoparticles can be used to enhance SPR signals and overcome the challenges related to the small molecule detection [98,99,100,101,102,103]. However, it could be argued that after incorporating a nanoparticle label to the sensor configuration, such a method is no longer genuinely label-free. Also, advanced SPR instrumentation based on microfluidic channels [104,105,106] or imaging surface plasmon resonance [107,108,109] have enabled multiplex detection of several targets simultaneously in the inhibition assay format.

### 4.2. SPR Sensors Based on Aptamers

Aptamers are rarely able to compete with monoclonal antibodies when it comes to affinity, but they show several advantages such as relatively simple synthesis without significant batch differences. Moreover, chemically synthesized aptamers are easily modified or extended from their 3’ or 5’ end, which allows designing particular immobilization strategies which do not have a negative influence on their recognition activity. Direct binding of thiolated aptamers to the gold surface is a widely used method, although many other alternatives have been reported as well. As with other recognition elements, the immobilization manner of aptamers is crucial as in some cases immobilization at one end of the aptamer has been seen to abolish the binding [110].

**Figure 4:**
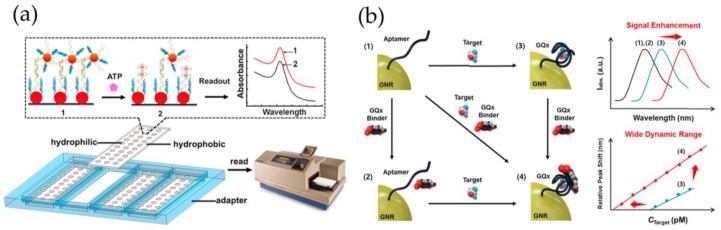
(**a**) Wall-less LSPR array chip for detection of adenosine triphosphate (ATP) using a normal microplate reader. Plasmonic nanoparticles (NPs) are immobilized on hydrophilic–hydrophobic patterned glass slide and a double-gold NPs system constitute a competitive replacement assay for signal amplification. (**b**) LSPR sensor for small molecule detection based on (1) aptamer-modified gold nanorods (GNRs), (2) addition of the G-quadruplex (GQx) binder, (3) addition of a target that induces GQx structure, and (4) addition of the target and GQx binder; interaction between the GQx binder and GQx occurs. GQx binder provides signal enhancement and enables a broad dynamic range. (**a**, reprinted from [113]; **b**, reprinted from [119], both with permission from Elsevier).

First aptamer-based SPR sensors were based on the competitive assay-format. For example, after an unsuccessful first trial to directly detect the small molecule target binding with an immobilized aptamer, neomycin B was covalently immobilized onto the sensor surface, and detection of free neomycin B was achieved in the competitive format with a range of quantification between 10 nM and 100 μM [111]. On the other hand, competitive replacement assays which take advantage of aptamers and partially complementary single-stranded DNA (ssDNA) have been reported as an alternative to improve the detection of small molecules. Hybridization of gold nanoparticle (AuNP)-tagged secondary ssDNA with the immobilized aptamer results in a substantial change in SPR signal, whereas upon target binding the secondary ssDNA is not able to hybridize with the target-bound aptamer causing a remarkable decrease in the signal. In this manner, adenosine was detected over the range of 10^–9^ to 10^–6^ M using an aptamer immobilized on SPR gold film and complementary ssDNA with AuNPs [112]. Alternatively, a double-AuNP system comprising of ATP (adenosine triphosphate) aptamer functionalized 39-nm-AuNPs array chip and a partially complementary ssDNA which was immobilized on 13-nm-AuNPs. A wall-less LSPR array chip was fabricated on a hydrophilic-hydrophobic patterned glass slide, which enabled high throughput detection and the chip fabrication and sample processing could be simplified using the wall-less configuration (Figure 4a). The detection limit was reported down to 0.01 μM ATP which was a 5-order-of-magnitude improvement over the non-enhanced setup [113].

Interestingly, the versatility of aptamers as recognition elements has been elaborated by use of two anti-cocaine aptamer subunits in an aptasensor where one subunit was assembled on gold surface and the second subunit was labeled with AuNPs. In the presence of cocaine, binding of both aptamer subunits resulted in electronic coupling between the localized plasmon of the AuNPs and the surface plasmon wave of the gold surface and a significant shift in the SPR spectrum was observed. The dissociation constant of the aptamer complex was determined to be (8.9 ± 0.4) × 10^–6^ M and a detection limit of 10^–6^ M for cocaine was reported [114].

Later, advances in the sensor technology and alternative immobilization methods have also enabled the direct detection of small molecules. For example, Zhu et al. [115] reported Biacore aptasensor for the detection of OTA using streptavidin as a cross-linker to immobilize a biotinylated aptamer. Linear detection range from 0.094 to 10 ng/mL of OTA with a lower detection limit of 0.005 ng/mL was reported, and after liquid-liquid sample extraction spiked wine and peanut oil samples were analyzed with recoveries from 86.9% to 116.5%. An alternative immobilization strategy on the Biacore sensor was reported by Chang et al. [116] who characterized the affinity and kinetics of a diverse panel of 12 small molecule-binding RNA and DNA aptamers. Instead of direct aptamer immobilization or streptavidin linker, a poly(T) DNA linker was covalently immobilized to a Biacore sensor chip, and the aptamer was captured through hybridization of a poly(A) tail, which enabled simple regeneration of the surface and testing different aptamers or targets on the same chip.

Recently, direct detection of tetracycline was reported with a Biacore sensor where the aptamer was immobilized on the top of a tetrahedron nanostructure to provide better accessibility of the target. The sensor was based on the conformational reorganization of the aptamer which formed G-quadruplex structure upon target binding. The aptasensor was validated in a real application for tetracycline screening in multiple honey samples, and the detection limit was calculated to be 0.0069 μg/kg, 10-fold lower than that of the aptasensor with the single-stranded aptamer [117]. Also, an LSPR-based aptasensor based on the G-quadruplex structure and gold nanorods (GNRs) enabled detection of OTA in nM-range [118]. Later, an enhancement in a similar approach was achieved using G-quadruplex (GQx) binders to increase the signal change upon target binding (Figure 4b). Addition of berberine as GQx binder improved the detection limits for OTA, ATP, aflatoxin B_1_ (AFB_1_), and potassium ions 1000-fold [119]. Recently, the same group reported another aptasensor for OTA detection based on an optical fiber coated with aptamer-modified GNRs which enabled in situ detection of OTA by simply dipping the fiber into a solution. Linear range was determined from 10 pM to 100 nM with a detection limit of 12.0 pM, and OTA-spiked samples were analyzed from 50% grape juice with recoveries from 85.5% to 116.9% (1–100 nM OTA) [120].

Examples of instrumental advances in aptasensors include the development of a lab-made plasmonic sensing platform based on sinusoidal gratings and the azimuthally-controlled SPR under phase interrogation which leads to enhanced refractive index compared to the classic grating-based SPR setup. After optimization of the aptamer immobilization strategy, the biosensor was demonstrated to detect down to 0.2 ng/mL of OTA with a detection limit of 0.005 ng/mL [121]. In a different approach, a portable, palm-sized transmission-localized surface plasmon resonance setup with aptamer-functionalized gold nanoislands deposited on a glass slide was reported for the detection of tobramycin, measuring concentrations down to 0.5 μM in buffer and down to 10 μM in filtered undiluted blood serum with a theoretical detection limit of 3.4 μM [122].

### 4.3. SPR Sensors Using Molecularly Imprinted Polymers

In recent years, MIPs have been rather widely used in SPR sensors for small molecule detection [32]. The interaction of the target molecule with the active sites of the polymer causes a change in the dielectric nature of the sensing layer which can be detected by SPR. Compared to antibodies, MIPs can provide a more robust surface as they can withstand better harsh regeneration conditions and are less susceptible to lose their binding capability [123]. Several methods to prepare a selective MIP layer on SPR sensor chips have been described, and they are either based on physical deposition or covalent coupling of the MIP film or particles onto the chip or, alternatively, involve in situ polymerization directly onto the sensor surface. For example, the spin coating has been used to create the recognition layer for SPR sensors with imprinted nanofilms or -gels for the detection amoxicillin [124], citrinin [125], and pesticides [126]. A recent report showed that an enhancement in the SPR signals was achieved using a nano-hybrid film for the detection of ractopamine [127]. In this work, MIP particles were synthesized by precipitation polymerization and coated with AuNPs and reduced graphene oxide to improve the SPR signals. The novel SPR sensor with the nano-hybrid film for ractopamine detection had a wide linear range from 20 to 1000 ng/mL with a detection limit of 5 ng/mL. Another recent work reported the use of atrazine imprinted nanoparticles which were deposited and dried onto the SPR chip. Attachment of the nanoparticles was confirmed by scanning electron microscopy, and the SPR measurements showed the linear response from 0.5 ng/mL to 15 ng/mL with a detection limit of 0.7134 ng/mL of atrazine [128].

Alternatively, imprinting can be performed directly in situ on the sensor surface thus avoiding a separate MIP immobilization, which is the approach that the majority of reported SPR sensors for small molecules rely on. Various methods for creating imprinted films have been reported, including thermal-initiated polymerization, photo-initiated polymerization, and electrical polymerization. For example, molecularly imprinted polypyrrole films were prepared via electropolymerization onto bare gold chips for the detection of zearalenone [129] and DON [94]. Likewise, π-conjugated MIP with nanopatterns for T-2 toxin was prepared on SPR chip by in situ electropolymerizations. Kinetic measurements showed excellent affinity with a *K*_D_-value of 12.7 fM, and the sensor had a linear response for T-2 toxin from 2.1 fM to 33.6 fM with a detection limit of 0.1 fM (0.05 pg/mL). Interference was studied with high concentrations of other toxic small molecules which showed less than 10% selectivity efficiencies; however, the response using other toxins with similar structures was not tested [130]. By surface-initiated polymerization, where the initiator is immobilized to the sensor surface prior to the polymerization, ultrathin MIP films have been prepared, for example, for DA [123], malachite green [131], acephate [132], ametryn [133], and profenofos [134] detection showing the potential of MIPs for selective and sensitive analysis of small molecules. Another SPR sensor based on nanoscale MIP film as recognition element was developed for selective detection of the antibiotic ciprofloxacin (CIP). The MIP film was prepared by in situ photo-initiated polymerization method, and the sensor had good linear relation with CIP concentration over the range 10^–11^–10^–7^ M and the detection limit was determined to be ~0.08 μg/L. Furthermore, using the MIP-modified SPR imaging (SPRi) chip with separate sensing spots, the SPR response for two different types of antibiotics (CIP and azithromycin) could be measured simultaneously [135].

MIPs are also able to provide a three-dimensional binding matrix which can improve the sensitivity due to higher binding capacity. For this purpose, a water-compatible macroporous molecularly imprinted film (MIF) was synthesized by photo copolymerization of monomers, cross-linker and polystyrene nanoparticles in the presence of testosterone as the template molecule [136]. After removal of the template and the polystyrene nanoparticles, a macroporous MIF was formed. By in situ polymerization, the thickness of the film could be observed as the shift in the SPR resonant angle and the UV radiation was stopped when, approximately, 177 nm thick film was observed, a thickness comparable to the penetration depth of SPR. SPR-based MIF sensor was applied to testosterone detection in buffer and artificial urine with a detection limit down to 10^−15^ g/mL (i.e., 3.5 fM) which is among the lowest values reported for small molecule detection by SPR. Conventional MIF showed 30-fold lower response compared to the macroporous MIF indicating improved accessibility and sensitivity for testosterone due to the high porosity. High stability of the MIF was proved as the sensor chips could be stored at room temperature for 8 months and, approximately, 84% of their affinity was retained, whereas conventional antibody-based biosensors survive storage at room temperature only for days, which highlights one of the most remarkable advantages of MIPs as recognition elements.

**Figure 5:**
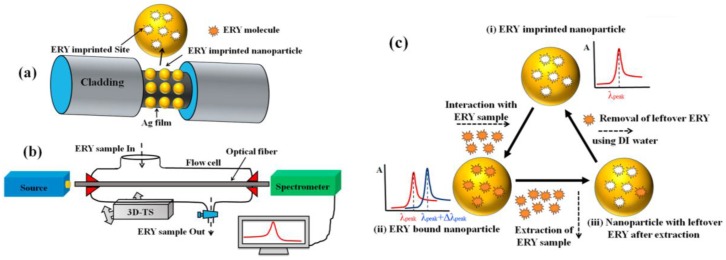
Schematic of (**a**) fabricated sensing probe for the detection of erythromycin (ERY) using the fiber optic core decorated with the coatings of silver and a layer of ERY imprinted nanoparticles (**b**) experimental set-up and (**c**) sensing mechanism. Figure reprinted from [142] with permission from Elsevier.

Additionally, MIPs have been used to create a sensing layer in fiber optic SPR sensors for which the cladding of a small part of the optical fiber is replaced by a thin metal film on top of which a sensing layer is constructed, and the evanescent waves generated at the core–metal interface are then used to excite surface plasmons at the sensing interface [62]. Application of MIPs to fiber optic SPR sensors has been reported to enhance their sensing ability for the detection of selected small molecules. For example for 2,4,6-trinitrotoluene (TNT) detection, an unclad plastic optical fiber was coated with a thin gold film where the selective MIP film was deposited and based on the changes in the obtained SPR transmission spectra, a detection limit of 5.1 × 10^−5^ M was established [137]. Similarly, a sensing probes for tetracycline [138], melamine [139], atrazine [140], profenofos [141], and erythromycin [142] detection were fabricated by coating the unclad core of an optical fiber with a 40 nm thick silver film which was further coated with the target-specific MIP by dip coating (Figure 5). A red shift in resonance wavelength indicated target recognition, and excellent detection limits for atrazine (LOD = 1.92 × 10^−14^ M), profenofos (LOD = 2.5 × 10^−6^ μg/L) and erythromycin (LOD = 1.62 × 10^−3^ μM) were reported. To improve the sensitivity, a 10 nm thick aluminum layer was added between silver and MIP layer and slightly lower limit of detection for atrazine was measured [140]. Erythromycin sensor was additionally applied to the analysis of spiked milk and honey samples, and excellent recoveries were reported for micromolar erythromycin concentrations [142].

Alternatively, enhancement in the sensitivity of MIP-based detection has been achieved by incorporating gold nanostars in LSPR sensor for the detection of TNT [143] and l-nicotine [144]. TNT-specific MIPs, identical to the previously reported [137], were used with five-branched gold nanostars and three times better sensitivity was obtained (LOD = 2.4 × 10^−6^ M). Further enhancement of the sensitivity was achieved using tapered optical fibers (LOD = 7.2 × 10^−7^ M), which was attributed to the reduction of incidence angles of the guided rays in the fiber close to the critical angle of the unclad uniform tapered region [143]. For ascorbic acid (vitamin C) detection, enhancement in the sensitivity of a polyaniline MIP-based optical fiber SPR sensor [145] was achieved by employing both SPR and LSPR techniques [146]. In situ, molecularly imprinted polyaniline-Ag nanocomposite was coated on the Ag layer on the optical fiber core thus combining LSPR of the Ag nanoparticles and SPR of the Ag thin film. Compared to the LSPR probe without the Ag film (LOD = 1.117 × 10^−10^ M), lower detection limit could be achieved with the LSPR+SPR probe (LOD = 7.383 × 10^−11^ M) and ascorbic acid contents of commercial vitamin C tablets could be analyzed with good recoveries [146]

### 4.4. Enzyme-Based SPR Sensors

Although for certain sensor applications enzymes are the most extensively studied recognition elements, only a few examples of enzyme-based SPR sensors have been described [147]. In some recent strategies acetylcholinesterase (AchE), a key enzyme in neurotransmission, which is inhibited by organophosphorus compounds such as nerve agents, pesticides and several toxins, has been used in SPR sensors for the detection of these inhibitors [148]. Fiber-based LSPR-sensor for pesticide detection using AChE covalently coupled to AuNPs was based on the change of the light attenuation in the presence of pesticides which inhibited the activity of AChE to hydrolyze acetylcholine chloride (ACh). In optimized conditions, the detection limit of the sensor was 0.234 µg/L and using a powerful nucleophilic agent the sensor surface could be reactivated with 94% recovery rate after six cycles of inhibition [149]. Based on the same principle, AChE was used in a fiber-optic sensor based on silver coated core of a plastic-cladding silica fiber. AChE was immobilized using gel entrapment, and detection of chlorpyrifos pesticide (CPF) was reported in the micromolar range [150]. More recently, Milkani et al. [151] reported direct detection of AChE inhibitors used for Alzheimer’s disease therapy by immobilizing AChE covalently to a self-assembled monolayer on the gold surface of a commercial SPR sensor. The reversible carbamate inhibitors, neostigmine and serine, could be detected at micromolar concentrations, and considering the small target size, a relatively large change observed in the refractive index was attributed to the conformational changes in AChE as a result of inhibitor binding to the enzyme’s active site. It has also been reported that conjugation of the target molecule to a carrier protein does not significantly alter the AChE recognition but could be used to increase the SPR signals. In the AChE-based SPR sensor by Puiu et al. [152], the target AFB_1_ alone did not give a detectable SPR signal, but using AFB_1_–horseradish peroxidase (HRP)-conjugate in a competitive approach an detection limit of 0.003 μM (0.94 ng/mL) for AFB_1_ was reported.

**Figure 6:**
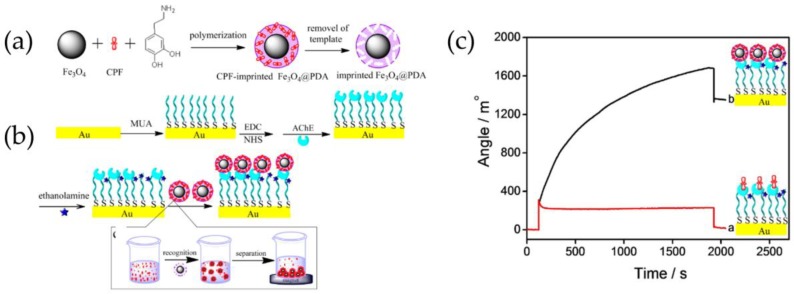
(**a**) Preparation of magnetic MIPs for chlorpyrifos (CPF) recognition and (**b**) schematic illustration of the stepwise preparation process of the sensor surface with immobilized AChE. (**c**) SPR response curve with 10 μM CPF using direct detection of free CPF in PBS (red) and amplification with magnetic MIPs (black). Figure reprinted from [153], copyright (2013) American Chemical Society.

Enhancement in AChE-based detection has been achieved using magnetic MIPs with recognition sites for pesticide CPF, which enabled enrichment of the target to the sensor surface. Magnetic MIPs were synthesized by self-polymerization of dopamine on the surface of Fe_3_O_4_ nanoparticles in the presence of the template CPF. Rebinding of the target and analysis of these CPF-imprinted nanoparticles by AChE-based SPR showed a low detection limit of 0.76 nM due to the significant signal amplification caused by the high molecular weight of the MIPs. Compared to direct analysis of CPF, approximately 64 times higher SPR angle shift could be observed with CPF-imprinted nanoparticles (Figure 6). Although CPF rebinding to MIPs required 12 h incubation, which can be considered as a limiting aspect, the MIP-based method showed excellent selectivity against other pesticides tested [153].

## 5. Surface-Enhanced Raman Spectroscopy

Surface-enhanced Raman spectroscopy (SERS) is a powerful vibrational spectroscopy technique that has unique features compared to other sensor devices using various kinds of signal readouts, including colorimetry, fluorescence, surface plasmon resonance, and electrochemistry [154,155,156]. SERS is a vibrational spectroscopy technique that can be used to identify unknown compounds since it provides structural information of the target molecule as a “fingerprint,” similar to that derived from infrared spectroscopy. Also, SERS sensors can be used to analyze aqueous-rich samples and complex matrices, such as food samples, because water does not interfere in SERS signals and it has deep penetration depth.

**Figure 7:**
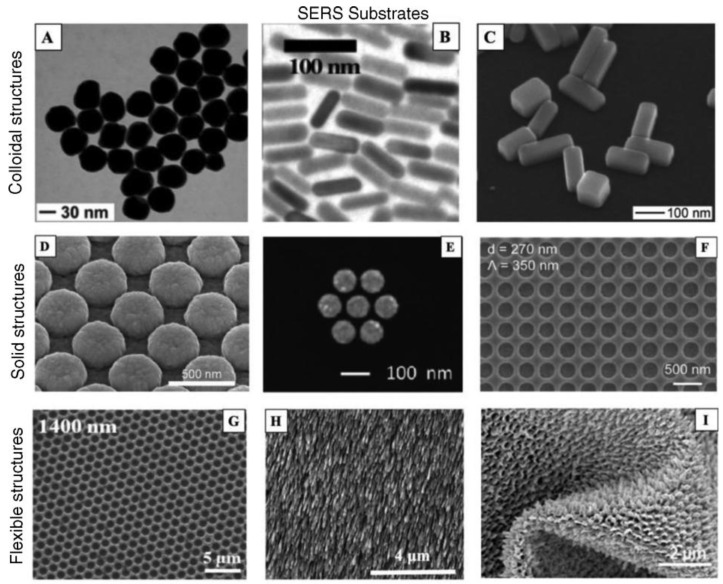
SEM images of different types of SERS substrates. (**A**) Spherical gold nanoparticles, (**B**) gold nanorods, (**C**) silver nanobar, (**D**) silver plasmonic nanodome array, (**E**) gold nanocluster, (**F**) gold nanoholes, (**G**) silver nanovoids, (**H**) silver nanocolumnar film, and (**I**) silver nano-pillars. Reprinted with permission from [158], copyright (2017) De Gruyter.

Generally speaking, Raman scattering is tremendously inefficient due to the small scattering cross section of ca. 10^–30^ cm^2^/molecule, which is about 14 orders of magnitude lower than the cross sections of fluorescent dye molecules [157]. Therefore, it is fundamental the use of SERS reporters or substrates that, similarly to SPR-based sensors, enhance the Raman signal to improve the sensitivity. Mostly all SERS reporters are plasmonic nanostructures, such as gold, silver, copper, or aluminum nanoparticles with dimensions lower than the wavelength of the incident light (Figure 7).

In these particular cases, the electronic oscillations are confined within a small volume, resulting in a specific type of surface plasmon called localized surface plasmon (LSPR). The excitation of LSPR plasmons produces a strongly amplified electromagnetic (EM) field because of the collectivity oscillation of the conductive electrons on the nanoparticle surface. The basic concept of surface-enhanced Raman spectroscopy is that the Raman scattering signal of molecules at or close to the metallic surface can be enhanced to a factor of 10^10^–10^11^ [156]. There are at least two enhancement mechanisms in SERS [159,160]: (1) electromagnetic, referring to the electromagnetic effect that occurs near to the particle surface and is associated with long-range enhancement; (2) chemical effect, induced by the charged transfer between the metal nanoparticle and the molecule chemically adsorbed.

**Figure 8:**
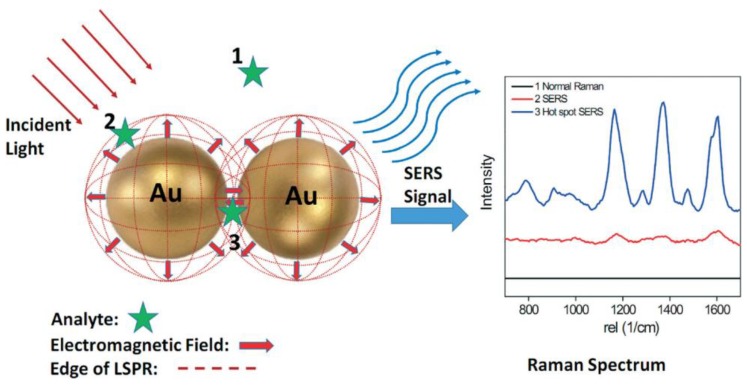
Schematic of SERS effect for a small molecule on gold nanoparticles (AuNPs). (1) Analyte outside the enhanced magnetic field (red dotted lines), no Raman signal is observed; (2) analyte located within the enhanced magnetic field but at long-distance, and (3) analyte located within a “hot spot”. Reprinted with permission from [159]. Copyright 2015 The Royal Society of Chemistry.

Several factors significantly influence SERS, such as the type and energy of the bond between the molecule and the nanoparticle (NP), the surface roughness of the NP, light excitation wavelength and media conditions (e.g., pH, buffer nature). Interestingly, a significant enhancement has been observed when the analyte molecules are located in the gap between two or more nanoparticles, or are situated at sharp angles or tips of anisotropic plasmonic NPs (e.g., rods, stars, prisms). These areas are known as SERS “hot spots” and maximization of their number is desirable (Figure 8). Although aggregation of metal NPs in liquid induces “hot spots”, reproducibility during measurements is challenging and a real disadvantage in sensing applications. Thus, part of the current interest in material science lies on the preparation of SERS reporters with three-dimensional large-ordered nanostructures to achieve signal uniformity and reproducibility. Some examples include rods, holes, clusters, domes 3D nanostructures which have been successfully fabricated (Figure 7) [158]. Moreover, the use of coating nanomaterials to improve the chemical stability of the metal structures and to preserve the SERS signal is under investigation. For example, graphene, mesoporous silica, polymers, and even proteins are starting to be widely used in the fabrication of SERS reporters [161].

Benchtop Raman microscopes have been the gold standard platform for analytical methods based on SERS [162]. However, the large size and high cost restrict their use to the laboratory, although the transition to the field as portable SERS sensors is a reality proved by the arrival of affordable small portable Raman spectrometers with dimensions similar to a smartphone, such as FirstDefender^TM^ RMX (Thermo Fisher Scientific, Inc., Waltham, MA, USA) [163]. The diversity of SERS-active platforms offers sufficient choices for analyzing proteins, disease biomarkers, ions, toxins, bacteria, viruses, etc.

In contrast to label-based SERS devices, in which the accuracy depends on the selective binding of the label to the target analyte, in label-free SERS methods the signal is directly dependent on either the analyte or the recognition element–analyte complex; thus understanding intra- and intermolecular interactions of the analyte is feasible. Direct recognition of the analyte by its characteristic Raman vibration bands when it is adsorbed, chemically or physically, on the SERS reporter surface is the most straightforward approach for detecting and quantifying small molecules. For example, dithiocarbamate pesticides thiram, ferbam, and ziram were detected individually using a gold nanoparticle colloid as SERS reporter [164,165]. The three organic pollutants were adsorbed firmly on the plasmonic surface through their sulfur groups allowing detection limits as low as 34 nM, 26 nM and 23 nM, respectively. Using a different SERS reporter type, Meng et al. [164] prepared a self-assembled layer of silver nanocubes on a flexible polyethylene film. Direct deposition of SERS sensor pieces on contaminated oranges demonstrated the presence of 10 nM of thiram, 1 μM of 3-chlorobiphenyl, and 10 nM of parathion.

A critical factor in label-free detection using SERS sensors is that the analyte should be confined inside the enhanced EM field of the SERS reporter. This issue is a significant challenge for SERS sensing since high non-polar or small hydrophobic molecules present low affinity for hydrophilic SERS reporters (e.g. Ag, Au, etc.), which prevents their detection at low concentrations. In this context, (bio)recognition elements can provide a set of analytical strengths that add value to SERS sensor platforms. For example, they can provide a degree of selectivity and an increase in the sensitivity by concentrating the analyte at the sensing surface. Most of the (bio)recognition elements used in label-free SERS sensors are antibodies, aptamers, molecularly imprinted polymers, and small molecules affinity agents. The following sections highlight label-free SERS sensor approaches reported for small molecule detection using the aforementioned (bio)recognition elements. Table 2 summaries some of the most interesting approaches from recent years.

### 5.1. SERS Sensors Based on Antibodies

The first report that described the use of antibodies as the biorecognition element in SERS was a label-based “sandwich” immunoassay. The target, thyroid stimulating hormone (TSH), was detected using anti-TSH capture antibodies bound to silver surfaces to form SERS probes and a detection antibody labeled with *p*-dimethylaminoazobencene, a well-known SERS reporter. The intensity of the resultant Raman scattering signal and the TSH concentration had a linear relationship ranging from 4 to 60 μIU/mL [166].

It is worth noting that, as in the case of SPR sensor platforms, the number of direct SERS methods for small molecule detection using antibodies is limited. This is likely due to the large size of the antibody (ca. 150 kDa) that occupies the volume just above of the SERS substrate area and thus, is in the most active region of the enhancing EM field [167]. Antibodies, which are composed of amino acids, produce a significant SERS signal particularly from the side chains of phenylalanine, tyrosine, and tryptophan residues [168], and thus, a discrepancy between the antibody and small molecule signal is produced. As a consequence, spectral information of the target small molecules can only be obtained under two particular circumstances: (1) binding of the small molecule to the antibody paratope triggers a structural change in the latter, or (2) the small target features a large scattering cross-section.

**Figure 9:**
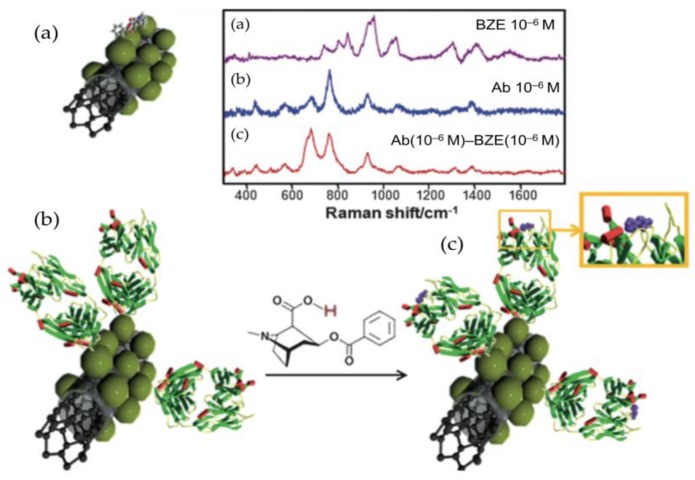
Schematic representation of benzoylecgonine (BZE) detection by SERS using silver-coated carbon nanotubes (CNT@Ag). Direct binding of BZE to AgNPs (green spheres) (**a**), label-free indirect detection of BZE on CNT@Ag coated with BZE-specific antibody fragment alone (b) and in complex with BZE (**c**). Corresponding SERS spectra are depicted in the graph on top. Figure reprinted from [169] with permission from The Royal Society of Chemistry.

A representative example of a direct SERS-based immunoassay for the detection of small molecule targets is the work of Sanles-Sobrido et al. [169]. The authors demonstrated that BZE could be detected and quantified by simple comparison of the antibody-BZE complex Raman spectra, enhanced by the use of silver-coated carbon nanotubes, with the spectrum of the antibody alone. For supporting this strategy, structural information of the anti-BZE antibody was illustrated through the study by single-crystal X-ray diffraction and computational modeling of the modifications in the fragment antigen binding (Fab) region upon binding to BZE (Figure 9). The detection limit for BZE was in the range of 1 ng/mL (ca. 1 nM), which was in the same order of magnitude than using other immunological methods. Moreover, the reported SERS-sensor could be suitable for in situ detection of BZE even in complex biological fluids, similarly as was reported later with the antibody-based SPR-sensor for the same analyte [65].

An exciting alternative to minimize the strong SERS signal produced by the antibody is the use of recombinant antibody fragments. Up to now, the most used antibody fragment type is scFv (single chain fragment variable, 25 kDa) which consists only of the V_H_ and V_L_ domains, joined by a peptide linker. Surprisingly, to the best of our knowledge, no reports have been published based on the combination of label-free SERS-based immunoassays and recombinant antibodies for small molecule detection.

### 5.2. SERS Sensors Based on Aptamers

Aptamers are small (usually from 15 to 60 nucleotides, or 5–20 kDa) DNA or RNA oligonucleotides that have specific affinity for a target molecule. Since 1996, when the first aptasensors were published, their sensing applications are on the rise with particular emphasis in SERS-based aptasensors [170]. Despite their simplicity, aptamers feature excellent binding constants, resulting from the three-dimensional architecture acquired upon binding to the target molecule through hydrogen bonding, Van der Waals, and/or electrostatic interactions. Aptamer immobilization onto SERS substrates does not result in random orientation since there are only two ends, 5’ and 3’, in its structure. The most popular immobilization approaches are based on [171,172]: (1) the use of thiolated aptamers which behave as ligands to form metal-thiolated complexes; (2) adsorption or π-π stacking interactions between the oligonucleotide bases of the aptamer and graphene-modified SERS reporters; (3) covalent linkage of amino-functionalized aptamers to carboxylic acid groups present on a particular substrate.

Mostly all label-free SERS platforms based on aptamers, detect small molecules using either the direct signal from the target or the target/aptamer complex. The latter approach is relatively simple to analyze since the assignment of the SERS vibration peaks of the DNA bases is fully covered in the literature [173]. One of the first reports combining aptamers and SERS detection demonstrated a label-free optical method for monitoring cocaine and platelet-derived grow factor [174]. Similarly to work presented by Sanles-Sobrido et al. [169] SERS spectra of the DNA aptamer, acquired before and after exposure to the target analyte, enabled detection of the conformational changes in the oligonucleotide induced by the formation of the aptamer–analyte complex. This observation was well correlated with circular dichroism spectroscopy. Direct monitoring of the target molecule using a SERS device was described by Barahona et al. [175]. The approach presented an aptasensor for monitoring the pesticide malathion using polymeric microspheres that were prepared with methacrylic acid and ethylene glycol dimethacrylated. Then, the beads were activated with 2-aminoethanethiol and decorated with AuNPs in which thiolated anti-malathion aptamers were immobilized. Characteristic peaks of malathion were observed in a region of the spectra free of DNA signal, and finally, 495 cm^–1^ (shift assigned to P-S stretching) was selected for quantification of the pesticide. The linear range was established between 3.3–33 μg/mL and the detection limit was 3.3 μg/mL with an RSD < 14%.

### 5.3. SERS Sensors Based on Molecularly Imprinted Polymers

To solve some of the problems that prevented the use of molecularly imprinted polymers in SERS sensing platforms [35], homogeneous MIP films with controlled thickness have been synthesized using methods like atom transfer radical polymerization [133] and reversible addition-fragmentation chain transfer (RAFT) [123]. Moreover, the development of core-shell nanocomposite imprinted polymers is of high interest when the material includes additional benefits, such as magnetic, luminescent, or metal core nanoparticles [176]. AuNPs are the most widely used noble metal colloid in the manufacture of sensors based on core-shell structures (Au@MIP) thanks to the advantages it offers, such as optimal electro-optical properties, biocompatibility, and ease of fabrication and functionalization.

Wulff and coworkers reported one of the first approaches of SERS chemosensor based on MIPs [177]. Imprinted polymer layers were prepared on SERS archive surfaces built with either gold or silver deposited films. As a proof of concept, (2*S*,3*S*)-(+)-di-*O*-benzoyltartaric acid or *N*-benzyloxy-carbonyl-(l)-aspartic acid were selected as templates. The functional monomer and crosslinker used were *N,N′*-diethyl-4-vinylbenzamidine and ethylenglycol dimethacrylate, respectively. The rebinding of the target molecules was performed with a 10 mM solution of the aspartic acid derivative in 0.1 M HEPES at pH 7.3 during 15 min. Under these conditions, the uptake was followed at 1007 cm^–1^ and normalized with the polymer band at 1615 cm^–1^. As the authors mentioned, this method was not suitable for direct monitoring of the target molecules, pointing out the need for robust methods to anchor MIP layers onto metal surfaces without losing the SERS enhancement. Nevertheless, the work definitively opened the door to the use of MIPs for the development of SERS chemosensors as have been demonstrated in recent years [178,179,180].

**Figure 10:**
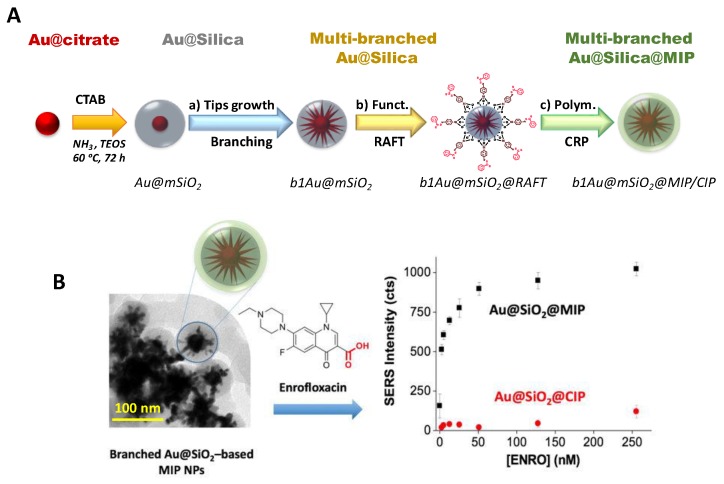
(**A**) Schematic representation of the nanocomposite fabricated in this work for the development of a SERS–MIP sensor, using Au@mSiO2 nanoparticles and a branching−functionalization−polymerization approach to produce branched Au@mSiO2@MIP/CIP (molecularly imprinted/control imprinted polymer) nanoparticles; (**B**) Schematic overview of the rebinding features towards the target molecule. Reprinted with permission from [181]. Copyright 2016 American Chemical Society.

To circumvent the aforementioned problem, Carrasco et al. [181] prepared selective Au@MIPs for ENRO using multibranched AuNPs as cores to act as intrinsic hot spots. The optimized nanostructures were obtained through a multistep synthetic approach that involved: (1) the growth of nanometric mesoporous silica layer, (2) formation of gold branches inside the silica, (3) functionalization with RAFT agent and (4) polymerization of the selective MIP (Figure 10). The use of this material resulted in a significant enhancement of the Raman scattering of ENRO upon binding to the selective sites of the MIP and improved sensitivity with a detection limit of 1.5 nM and minimal cross-reactivity toward potential interfering species.

In contrast to the numerous examples of MIP-metal composites prepared by core–shell approaches, Kamra et al. [182] prepared MIP microspheres by RAFT polymerization, templated to the target nicotine and decorated with terminal thiol groups to increase the adsorption to gold-based substrates. Raman signal of nicotine was enhanced by positioning the SERS substrates near the specific sites of the MIP by three different strategies: (1) coating MIP microspheres with gold colloids, (2) sputtering of AuNPs onto the MIP surfaces, and (3) measuring the MIP microspheres trapped on a commercial SERS-active substrate. All the approaches tested transformed the MIP microspheres into SERS-active substrates although gold colloidal coating yielded the strongest signal when nicotine was detected.

In a different strategy, Ashley et al. [183] combined the separation properties of magnetic molecularly imprinted polymer (MMIP)-based sample pretreatment with SERS detection for quantitative analysis of cloxacillin in pig serum. Magnetic FeO_x_ nanospheres were synthesized and coated with mesoporous silica and metacryloxy propyl trimethoxyl silane to allow the polymerization of the MIP layer directly attached to the silica surface. The target antibiotic was then extracted from pig serum using the MMIPs and eluted into a clean solvent. Then, the extract was deposited into the SERS substrates, consisting of vertical silicon nanopillars coated with gold, and quantitative analysis was performed using acetic acid as internal standard. The sensor presented good sensitivity (7.8 pmol) toward the antibiotic with recoveries ranging from 85% to 126%. Although the results were adequate, the strategy followed for measuring rendered SERS redundant because of the MIP specificity and the separation of the target from the matrix by extraction.

The multiplexing ability of SERS sensing in combination with MIPs was demonstrated by Holthoff et al. [184] for the simultaneous analysis of TNT and their derivatives. The MIP against TNT, based on organosilane precursors, contained 3-mercaptopropyl trimethoxysilane to facilitate the adhesion of the spin-coated MIP xerogel to the SERS reporter through thiol-metal interactions. The MIP bound selectively dinitro species that could be differentiated by the molecular “fingerprint” of each analyte afforded by the SERS measurements. Quantification of TNT was performed at 1352 cm^–1^, the peak corresponding to the asymmetric stretching band for nitrate, presenting a detection limit of 3 μM in solution after 24 h exposure.

### 5.4. SERS Sensors Based on Molecular Traps

Small molecules are exciting alternatives to trap hydrophobic organic targets that have a very low affinity to SERS surfaces. The two most common molecular trap agents are partition layers and functional monolayers of small molecules [167]. For the first type, diffusion enables the transport of a set of analytes that are soluble inside of them. The second interact directly through covalent binding, hydrogen bonding, ionic, polar, nonpolar, etc.

**Figure 11:**
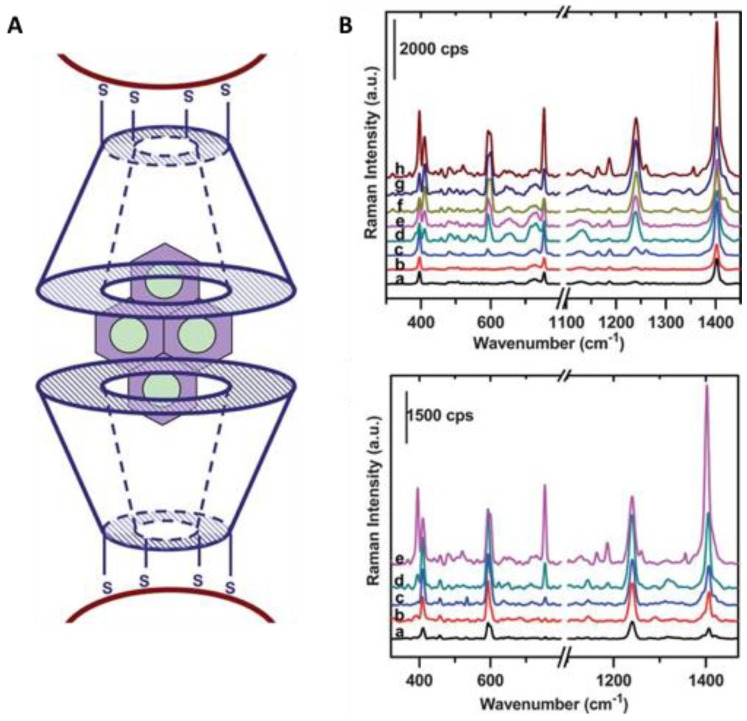
(**A**) Schematic representation of the host-guest complexation mechanism for pyrene. (**B**) Top graph: SERS spectra of mixtures of 333 mM anthracene with different concentrations of pyrene; a–h (0, 17, 25, 33, 83, 166, 250 and 333 mM). Botton graph: SERS spectra of mixtures of 333 mM pyrene with different concentrations of anthracene, from a–e (0, 83, 166, 250 and 333 mM). Reprinted with permission from [187]. Copyright 2010 the Royal Society of Chemistry.

For example, polychlorinated biphenyls [185] and polycyclic aromatic hydrocarbons (PAHs) [186] have been detected using partition layer-functionalized SERS substrates. For instance, decanethiol on silver substrates could perform pM sensitivity with multiplexing analysis capability. PAHs are a group of small molecules intensely studied using label-free SERS sensors and multicycle organic compounds, such as calixarenes, viologens, and cyclodextrins, have been broadly used [187,188]. For example, calix[4]arene dithiocarbamate [189] was able to host pyrene, benzo[c]phenanthrene, triphenylene, and coronene facilitating their SERS detection (LODs from 100 pM to 10 nM) when the calixarene was linked to AgNPs. Viologen dications (VGDs) lucigerin, diquat, and paraquat have also been evaluated as PAHs-molecular traps after their immobilization onto AgNPs [188]. VGDs were able to induce the formation of hot spots to locate the analyte yielding a significant intensification of the Raman emission of the target molecule. Lucigerin provided the most robust VGD–AgNPs sensor due to its bifunctional nature and its large aromatic character and allowed a limit of detection down to 10^–9^ M for pyrene detection. Another molecular trap used to determine PAHs (anthracene and pyrene) was reported by Xie et al. [187]. The designed system (Figure 11) used AgNPs modified with per-6-deoxy-(6-thio)-β-cyclodextrin. The SERS signals for each PAH were easy to differentiate and were significantly enhanced thanks to the selective cyclodextrin cavity that hosted the hydrophobic molecules near to the EM field. However, the sensitivity of the sensors was not impressive, reporting a limit of detection for anthracene of 10 μM and 7.5 μM for pyrene.

## 6. Interferometric Biosensors

Interferometric biosensors are based on refractive index changes produced by a biorecognition event which takes place in the sensing area. This change is measured with the aid of two light beams, the sensor, and reference beams, which are combined to produce an interference pattern. The sensor area is functionalized with the recognition element (e.g., antibodies, aptamers, enzymes) which is responsible for the selective recognition of the target analyte, and the immobilization of the recognition element is of crucial importance for the sensor performance [195,196,197]. On the other hand, a good intrinsic reference channel is necessary to obtain high sensitivity [195]. The reference channel is designed to take into account the background signals derived from thermal drift, the light source stability, mechanical noise, and non-specific binding, i.e., all the processes that also occur in the sensing channel other than the specific binding event [198].

**Figure 12:**
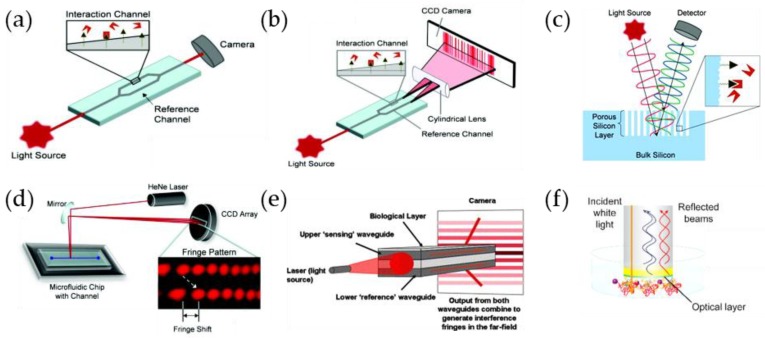
Schematic of the most commonly used interferometric configurations: (**a**) Mach–Zehnder interferometer (MZI), (**b**) a Young interferometer (YI), (**c**) porous silicon sensor, (**d**) backscattering interferometry (BSI), (**e**) dual-polarization interferometer (DPI), (**f**) biolayer interferometry. ((**a**–**d**), reprinted with permission from [201], copyright (2012) American Chemical Society; (**e**), reprinted wit permission from [214] and (**f**), reprinted with permission from [215] copyright 2004 and 2017 Elsevier.

Numerous interferometric configurations have been reported for measuring small molecules; the most used ones are described hereafter and shown in Figure 12. The two most commonly used platforms are the Mach-Zehnder (MZI) and Young’s (YI) interferometers which are both formed by a waveguide which splits a laser beam into the sample and reference arms and differ only in the way the two beams are merged to form the interference pattern. YI converges through natural divergence and MZI through a physically forced combination [196]. Dual polarization interferometer (DPI) employs two stacked planar waveguides (sample and reference) that are illuminated by a single laser beam, and the interference pattern is formed in the far field by emerging light [199]. Backscattering interferometry (BSI) differs from other interferometric techniques by its ability to measure interactions in solution within a channel or capillary [200]. Porous sensors use the Fabry-Perot interferometer principle where the interference is produced by reflected light from different surfaces within the sensor [201]. In biolayer interferometry (BLI) the binding event takes place at the end of an optical fiber where the reference is formed by a portion of propagated light that is reflected back towards the light source as it reaches a polymeric layer near the tip. The interference occurs between the reference beam and a small amount of light reflected from the interface between the fiber and the sample [202]. Along with these setups, there are several other interferometric configurations, such as reflectometric interference spectroscopy (RIfS) [203], spectral-phase interferometry (SPI) [204] or spectral-correlation interferometry (SCI), among others. Moreover, some configurations can be found in commercial devices such as ForteBio Octet (BLI) or Optiqua MiniLab (MZI).

Interferometric sensors can be produced in optical fibers or integrated optical waveguides. Generally, optical fibers can avoid problems related to channel blocking [205] and enable manufacturing of compact and economical equipment [206], but integrated optics offer greater flexibility in the geometry of the design and the combination of materials, allowing greater complexity and ease of access to the optical path in evanescent field sensing [197]. Sensing surfaces are generally prepared by depositing on the surface of a glass substrate an optically transparent thin layer of a high refractive index, such as silicon nitride (Si_3_N_4_) [207,208] or tantalum pentoxide (Ta_2_O_5_) [209,210]. Light can be coupled into the waveguide either by prism coupling, end firing, or diffraction grating, the latter being the most commonly used in interferometry measurements due to the simple manufacturing in the waveguide by etching the surface or by embossing. [195].

Interferometric biosensors can monitor directly small changes in the optical properties and allow measuring binding and dissociation events in real time. These biosensors generally do not require prior sample treatment or additional steps such as washing. Consequently, they have been used in a broad range of applications such as sensing, determination of biomolecule or protein-ligand interactions [211], drug discovery [212], and kinetic measurements [202,211], among others. Also, multi-analyte or multi-sample analysis is possible by joining different channel pairs (sensing-reference) into a single waveguide [207,213]. Detection of small molecules with interferometric biosensors using different recognition elements is discussed in the following sections, and some representative examples are listed in Table 3.

### 6.1. Antibody-Based Interferometric Sensors

Only a few examples of antibody-based interferometric sensors for small molecules have been reported using the direct assay format. A polarization interferometer biosensor based on a planar waveguide for the detection of mycotoxins AFB_1_ [216] and OTA [217] was able to detect the mycotoxins at concentrations down to ppts in a direct immunoassay format. The operating mode was similar to MZI, but the design was simpler, and the waveguide was not split into two arms. The waveguides consisted of a thin Si_3_O_4_ layer (200 nm) sandwiched between thicker SiO_2_ layers (3 μm) and the light propagated in the core at an angle of 47° and experienced about 3000 [216] or 800 [217] reflections per mm. For biosensor development, a portion of the SiO_2_ layer was etched, and a layer of positively charged poly-allylamine hydrochloride was deposited, followed by adsorption of protein A molecules, negatively charged, followed by immobilization of the monoclonal antibodies selective to the analyzed mycotoxin. The liquid sample was placed in contact with the sensing window using a reaction cell that included inlet and outlet tubes. Any changes in the refractive index or in the thickness of adsorbed molecules on the Si_3_O_4_ layer affected the *p*-component of the polarized light while the *s*-component was used as a reference. The light was collected with a charge-coupled device (CCD) array, and the phase shift between *p*- and *s*-components of polarized light was converted to variations of light intensity using a polarizer placed in front of the CCD camera. The sensor detected 0.01 ng/mL of AFB_1_ or OTA in water.

Most commonly antibody-based interferometric sensors use the competitive immunoassay format with an immobilized the antibody [207], or the binding inhibition assay where the target [218] is attached to the surface (Figure 1). In some cases, structural analogs of the target compound are needed for immobilization if the analyte lacks appropriate groups required for covalent coupling [219,220,221,222,223], or due to the risks associated with the analyte [224]. Alternatively, a protein-conjugate can be immobilized on the surface. For example, Pagkali et al. [225] reported detection OTA in beer with an MZI where the sensing area was functionalized with OTA–OVA conjugate. This 10-channel sensor was based on a binding inhibition assay with signal amplification using a secondary antibody to form another bimolecular layer increasing the effective RI. The assay exhibited a LOD of 2.0 ng/mL and a dynamic range from 4.0 to 100 ng/mL in beer samples. Later, the authors used the same platform and methodology for simultaneously detect AFB_1_, fumonisin B_1,_ and DON, achieving LODs of 0.8, 5.6 and 24 ng/mL, respectively, in undiluted beer in less than 15 min [213]. In a different approach, Maragos [205] applied a BLI system for the detection of DON in wheat flour with using immobilized DON–BSA obtaining a detection limit of 0.1 mg/kg [226]. To improve the detection limit and assay time, they tested the use of a secondary antibody marked with HRP, as well as a primary antibody conjugated to colloidal gold. After signal amplification using colloidal gold, the detection limit was decreased to 0.09 mg/kg.

Given the importance of the suitable surface for the covalent binding of the analyte, Krieg et al. [210] compared the performance of amino-dextran and diamino-polyethylene glycol as biopolymer coatings for sensing purposes. After binding dimethylated amitriptyline, binding inhibitions assays were carried out with RIfS, and better results were obtained with the amino-dextran surface. The most sensitive assay showed a limit of detection of 268 ng/L in buffer and 540 ng/L in diluted (1:10) human serum, making it competitive even with HPLC measurements. Moreover, the platform showed high stability as 80 measurement cycles could be performed on each transducer chip after guanidine hydrochloride regeneration [220]. Recently in a different approach, using bimodal waveguide interferometry (BiMW), Chocarro-Ruiz et al. [219] employed several silanization protocols to evaluate the most suitable biofunctionalization procedure for Irgarol 1051 detection.

**Figure 13:**
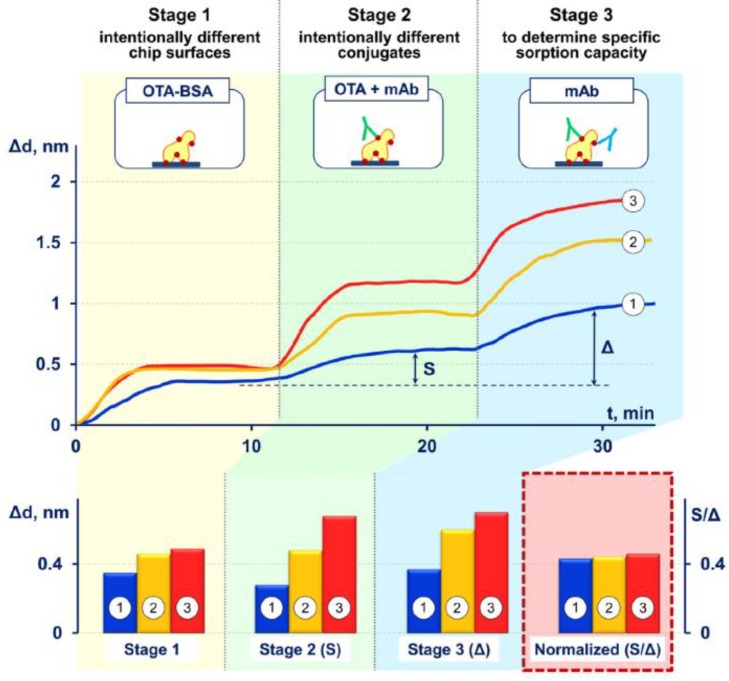
Scheme of reproducibility enhancement of label-free detection of small molecules. Spectral-correlation interferometry (SCI) sensograms for detection of ochratoxin A (OTA, 10 ng/mL) using intentionally different chip surfaces with different conjugates (top). Signal variations at each stage and the normalized signal (bottom). Reprinted with permission from [227]. Copyright 2017 Elsevier.

To improve the analytical characteristics of a porous silicon sensor, Orlov et al. [227] described a method for enhancing the reproducibility of the assay using the specific absorption capacity within the sensing area. For this purpose, after obtaining the signal produced by the competitive assay (S), an excess of the monoclonal antibody was put in contact with the surface to saturate the sites not occupied in the previous stage (∆). The normalized S/∆-ratio provided a better reproducibility even with non-uniform surface coatings (Figure 13). OTA, zearalenone, and AFB_1_ were detected in white wine with detection limits of 0.25, 0.48 and 1 ng/mL respectively, with CVs less than 5% and 2–3-fold lower detection limit compared a standard competitive assay. Furthermore, Tinsley-Bown et al. [224] develop a competitive assay for TNT detection which combined porous silicon interferometry with an Orthogonal Subspace Signal Projection Algorithm (OSPA), reaching a detection limit of 1 μg/mL. The OSPA method used reference reflectivity data before the sensing event to “self-calibrate” and measured any changes between the reference data and the test data overcoming the baseline drift, temperature fluctuation, and some systematic tendency such as the systematic reduction in optical thickness through erosion of the material.

### 6.2. Interferometric Sensors Based on Aptamers

**Figure 14:**
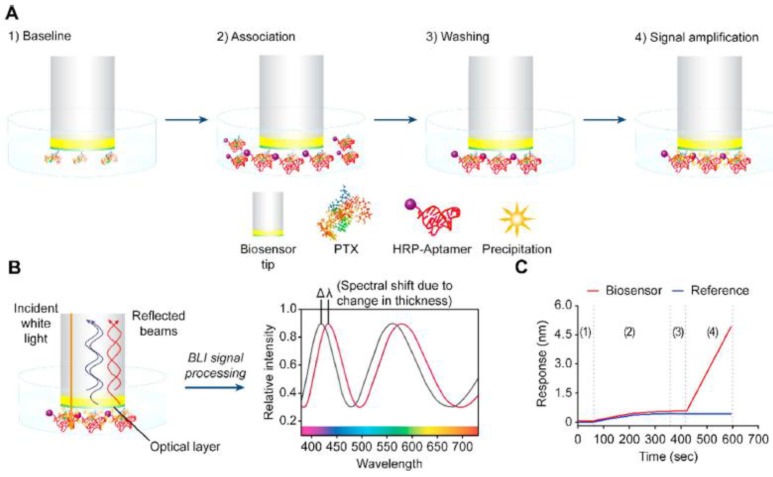
(**A**) Schematic of the working principles of the biosensor with the competitive assay for palytoxin (PTX) detection using horse-radish peroxidase (HRP)-modified aptamer. (**B**) Schematic of the biolayer interferometry (BLI)-based detection system and (**C**) the expected sensor response after each step: (1) baseline (1 min), (2) capture of free HRP-aptamer (5 min), (3) washing (1 min), and (4) signal amplification (3 min). Reprinted with permission from [215]. Copyright 2017 Elsevier.

Since aptamers first emerged in 1990 with the SELEX (systematic evolution of ligands by exponential enrichment) technique [228], they became an alternative to antibodies. However, they are still consolidating their role as biorecognition elements. SELEX process screens random sequences of a fixed length of a large oligonucleotide library; sequences are exposed to the target, and those with higher affinity are enriched through successive rounds. Wang’s group used this tool to find the aptamers with better affinity and specificity to different marine toxins, such as gonyautoxin 1/4 (GTX1/4) [229], nodularin-R (NOD-R) [230], and palytoxin (PTX) [215], and afterward applied to BLI sensors. Aptamers against GTX1/4 and NOD-R were employed in direct assays with detection limits of 50 pg/mL and 167 pM, respectively. BLI biosensor for PTX detection was based on a binding inhibition assay with immobilized PTX and an HRP-conjugated aptamer. After signal amplification by submerging the sensor tip in a 3,3′-diaminobenzidine solution, which resulted in the formation of a precipitated polymeric product directly on the biosensor surface, a detection limit of 0.04 pg/mL was obtained (Figure 14). In a similar approach, marine toxin saxitoxin (STX) was detected with a detection limit of 0.5 ng/mL and a linear range from 100 to 800 ng/mL [231] using a modified aptamer which had been improved via site-directed mutation and truncation and had a 30-fold higher affinity for STX compared with the parent aptamer [232,233].

Unlike in SPR where thiolated aptamers are often directly immobilized to the gold surface, interferometric sensors usually require a modified surface for the immobilization. The most widely used method, and perhaps the fastest, is the use of biotinylated aptamers in combination with surfaces modified with streptavidin [229,230,234] or avidin [235,236]. For example, bisphenol A (BPA) -specific aptamer was immobilized on the surface of a DPI sensor chip through biotin-avidin interaction, and detection limits of 2.5 mM on the mass signals and 1.7 mM on the thickness signals were reported [235]. In an alternative approach, adenine-rich ssDNA aptamer was immobilized through a preadsorbed layer of poly(ethylenimine) for the direct detection of Coralyn on a DPI. The mass, thickness and refractive index parameters were used to establish each calibration equation for the biosensor, achieving detection limits of 0.22 μM, 0.14 μM, and 0.32 μM respectively. Coralyn was detected in the range of 0.5 to 12 μM, with a high selectivity that was confirmed by comparison with three other common intercalators (ethidium bromide, daunomycin, and methylene blue) [237].

An unconventional competitive assay for testosterone was described by Zhang et al. [234] who used a BLI sensor with aptamers and testosterone-binding RepA protein. RepA could bind the biotinylated double-stranded DNA aptamers immobilized onto a streptavidin-coated chip in the absence of testosterone, whereas the conformational changes in RepA upon testosterone binding led to the displacement of RepA from the surface-bound aptamers. Testosterone could be determined in 17 min with a linear range of 2.13 to 136.63 ng/mL, exhibiting a sensitivity comparable to that achieved by HPLC.

Alternatively, using BSI which does not depend on surface-immobilization, Kammer et al. (2014) characterized interactions between small molecule and aptamers, providing *K*_D_ values with minimal sample manipulation on small volume sample quantities and at low nanomolar sensitivity. For this propose they measured the aptamer affinity for BPA (20.2 ± 2.1 nM), tenofovir (9.0 ± 1.4 nM) and epirubicin (626 ± 121 nM); showing values reliable with those published previously for the same molecules. In addition, *K_D_* values for aptamers to ampicillin (402 ± 99 nM), tetracycline (2.94 ± 0.28 mM) and norepinephrine (188 ± 64 nM) were determined [202].

### 6.3. Polymer-Based Interferometric Sensors

Also, MIPs have been described as bioinspired receptors for interferometric sensors [208]. MIPs can be produced directly on sensor surface [238], by thermal or photochemical initiation, or be integrated later by dip or spin coating [208,209]. In situ polymerization circumvents a separate immobilization step but sometimes has the disadvantage of long response times, low reproducibility and long-term stability, and regeneration problems.

Belmont et al. [208] applied two different methods for the deposition of MIPs, selective to atrazine, on the RIfS transducer surface. The first method consisted of in situ polymerizations of a thin film produced after spin-coating of monomers and template. In the second method, MIP nanoparticles produced by mini-emulsion polymerization were auto-assembled with the aid of polyethyleneimine. Using the auto-assembled particle film in a RIfS platform, atrazine detection was established at concentrations as low as 1.7 ppm which was several orders of magnitude lower than previously reported with the same surface using a chiral chemosensor [238] for (*R*,*R*)- or (*S*,*S*)-2,3-di-*O*-benzoyltartraric acid. RIfS has also been used for the detection of L-Boc-phenylalanine anilide through miniemulsion polymerized imprinted nanospheres which provided a detection limit of 60 µM, good reproducibility, and selectivity, as well as long-term stability for up to 1 year [209].

Also, non-imprinted polymers have been used as recognition elements in interferometric sensors. Porous silicon (pSi) films, which can be fabricated with the desired morphological structures, allows the design of a porous matrix to optimize the access of the target analyte, making it suitable for sensing applications [239]. These surfaces can be combined with polymers to carry out selective recognition, as in the glucose sensor described by Krismastuti et al. [240] using phenylboronic acid polymers. Thiol-terminated poly(4-vinylphenylboronic acid) was covalently bound to the pores of succinimidyl 4-(*p*-maleimidophenyl)butyrate modified pSi. In the presence of glucose, the properties of the polymer changed as cyclic boronate esters were formed. Surface behavior was monitored by interferometric reflectance spectroscopy (IRS) which could detect as low as 0.15 mM glucose at physiological pH, and no interferences were found when measuring in wound fluid samples.

A less common form of detection is based on measuring the decrease in surface thickness caused by polymer degradation. Wu et al. prepared a dithiothreitol (DTT) sensor based on an optical fiber Fabry-Perot interferometer (FPI). When DTT solution was in contact with the sensor, a cleavage in the disulfide-crosslinked PAAm hydrogel on the optical fiber was produced and monitored in real time as changes in the refractive index. A concentration of 50 µM DTT was successfully detected within 36 min [241], improving the sensitivity 2000 times compared to previous work [242].

Finally, there is no clear difference between interferometry and diffraction because of the similarity between their principles from a physical point of view [243]. For example, antimicrobial ENRO could be detected via optical diffraction using a selective MIP on a Si_3_N_4_/SiO_2_ strip waveguide sensor. ENRO produced large signal response, saturating at 70 M and selectivity was evaluated with the temple analog flumequine [208]. Optical diffraction in combination with MIPs has also been used for testosterone detection. This approximation used a holographic structure (diffraction grating) obtained via photopolymerization of photoinitiators and monomers with interfering laser beams and was able to detect testosterone between 1 and 100 µM [244].

### 6.4. Protein-Based Interferometric Sensors

**Figure 15:**
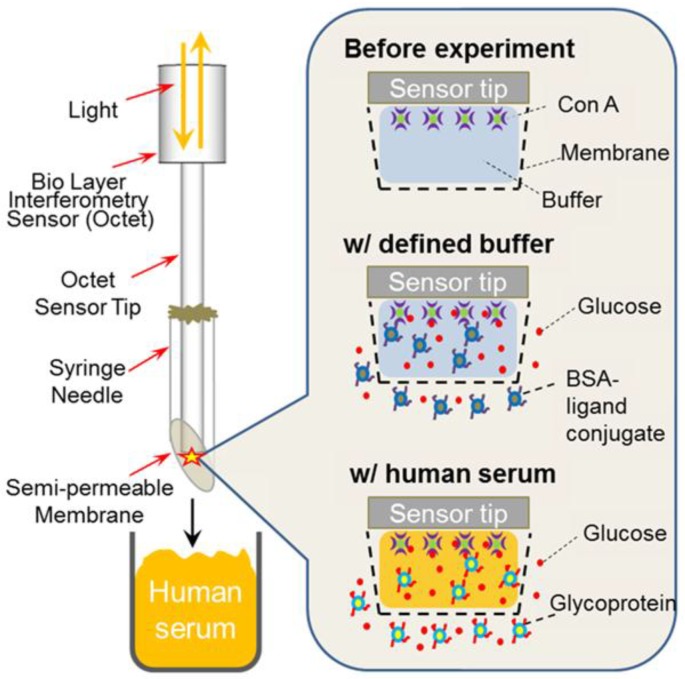
Schematic of the needle-type sensor. A syringe needle was modified and covered with a semi-permeable membrane to fabricate the needle-type sensor. Sensor tip was modified with concanavalin A (ConA) and bovine serum albumin (BSA)-ligand conjugate was kept outside of the semi-permeable membrane. Reprinted with permission from [246]. Copyright 2016 MDPI.

Although enzymes are used in numerous bioassay formats, there are hardly any examples in the case of interferometry [208]. Instead, certain models of interaction between small molecules and proteins have been used, such as glucose and mannose with lectin concanavalin A (ConA). An interferometric sensor developed by Paek et al. [245] used a semi-continuous determination of glucose in human serum by placing an optic fiber in the hollow of a syringe needle. The BLI glucose sensor measured glucose levels by quantifying the wavelength shifts caused by the binding of glucose to ConA immobilized on the tip. Direct glucose measurement was limited by the signal-to-noise ratios and to overcome this problem authors used a sugar-protein conjugate enclosed inside the needle by a semi-permeable membrane to competitively bind to the immobilized ConA and satisfy the clinical range of interest (70–200 mg/dL). Recently, the same group described a modification to the previous sensor because of considerable migration distance of the ligand to the sensor surface and the limited pore size of the membrane due to the artificial conjugates. Authors addressed these limitations by placing the sugar-protein conjugate outside of the membrane (Figure 15) and increasing the pore size. Results obtained with this platform were compared with the ideal BLI, showing high reproducibility and good stability over time, ascribed to the fact that the sensing zone was not directly exposed to serum and thereby avoided non-specific binding of other compounds [246].

BSI has also been used to study the binding affinity of mannose and glucose to ConA under significantly different physical conditions (tethered ConA on the surface and free solution). When comparing the results of the free solution with those obtained by having the ConA anchored at different distances from the surface, they observed that the binding affinity increased when the distance from the ConA to the surface decreased; thereby understanding how protein immobilization affects binding [211].

Alternatively, optical coherence tomography (OCT) has been used to study the viability of semi-noninvasive continuous monitoring sensors. The OCT sensor described by Ballerstadt et al. [247] for glucose measurement in interstitial fluid (ISF) was established using a porous membrane with a suspension of macroporous hydrogel particles and ConA within. A reference compartment, containing buffer or ISF, was added next to the glucose-sensitive compartment for signal normalization. Performance of the sensor was demonstrated in vitro for 160 days with excellent reversibility, stability and good response over the physiological concentration range (2.5–20 mM glucose). Additionally, to mimic in vivo conditions and evaluate the possibility of using the sensor as an implant under the skin, they covered the sensor with a tissue phantom and were able to measure glucose changes of 10 mM.

## 7. Conclusions

During the last decades, biosensors have become an essential tool for small molecule analysis in different disciplines, such as drug discovery, environmental security, and food safety, complementing and, in some cases, even replacing the existing analytical methodologies. Biosensors can provide not only selective and sensitive detection but can be also portable and offer a rapid response in real time, which makes them ideal for many applications. The performance of biosensors depends on many factors including, the bioreceptor layer, the transducer, and the instrumentation. Therefore all these different parameters must be taken into account in the design of the device.

SPR continues its reign as the most used label-free sensor configuration for small molecule analysis, partly due to the availability and simple use of advanced commercial devices. Nevertheless, significant advances in other transduction schemes have made important contributions to the field of label-free sensing. On the other hand, although antibodies are probably the most common biorecognition elements, during the last decade also several exciting applications using, for example, aptamers or MIPs as recognition elements have been reported. Table 4 collects examples of different optical label-free methods for the detection of OTA which has been one of the most widely used targets in the papers revised for this review.

Nevertheless, it should also be noted that the research in many label-free methods is still their infancy and many reports in the literature, particularly those related to instrumental or material improvements, are rather fundamental including only proof-of-concept bioassays and are thus yet to encompass the challenges associated with the real analyte analysis. For example, many of the most recent biosensors reviewed here have shown excellent characteristics for synthetic samples, but still, lack characterization in complex matrices which better resemble the real applications. Moreover, many designs based on nanostructures could be able to overcome some of the optical and biological limitations of sensors but the inherent nanofabrication procedures, such as electron beam lithography and focused ion-beam milling, might limit their widespread application [61]. In any case, as discussed in this paper, recent advances in the field of optical label-free methods are impressive, and new technologies and materials for improved detection are reported every year.

## Figures and Tables

**Table 1:** Examples of SPR- and LSPR-based methods using different recognition elements. The limit of detection (LOD), calculated as the blank response plus three times the standard deviation, is reported. Alternatively, the measured concentration range is shown between brackets.

Analyte	Recognition Element	Assay Format	Immobilization	LOD	Sample Matrix	References
Tetrodotoxin	MAb	Direct	Covalent amine coupling on CM5/CM7 sensor chip	0.091 ng/mL	–	[69]
Benzoylecgonine	MAb	Direct	Covalent amine coupling on a polycarboxylated hydrogel matrix	6.7 nM (=2 μg/L)	Oral fluid	[65]
Estradiol	MAb	Competitive inhibition	E2-BSA on the SCN-modified “charged” Au surface weak electrostatic interactions	[0.1–1000 ng/mL]	–	[86]
Tetracycline	Aptamer	Direct	DNA nanostructure	0.0069 μg/kg	Honey	[117]
OTA, AFB_1_, ATP	Aptamer	Direct, signal enhancement with berberine	Thiol modified aptamer on GNR	0.56 pM (OTA), 0.63 pM (AFB_1_), 0.87 pM (ATP)	–	[119]
Ractopamine	MIP/GNPs/rGO nano-hybrid	Direct	Deposition by spin-coating	5 ng/mL (LR 20–1000 ng/mL)	–	[127]
T-2 toxin	MIP	Direct	In situ electropolymerization	0.1 fM (=0.05 pg/mL) (LR 2.1–33.6 fM)	–	[130]
Testosterone	Macroporous MIF	Direct	In situ photo copolymerization	10^–15^ g/mL	Urine	[136]
TNT	GNS-MIPs	Direct	Polymerization on exposed POF core	7.2 × 10^−7^ M	–	[143]
Neostigmine Eserine	AChE	Direct	Covalent attachment of AChE to COOH-terminated SAM on Au surface	[10–1000 μM]	–	[151]
Chlorpyrifos	AChE/MIP	Direct after MIP capture	Covalent attachment of AChE to COOH-terminated SAM on Au surface	0.76 nM (LR 0.001–10 μM)	–	[153]

Abbreviations: AChE, acetylcholinesterase; aflatoxin B_1_, AFB_1_; ATP, adenosine triphosphate; DR, dynamic range; GNP, gold nanoparticle; GNR, gold nanorod; GNS, gold nanostar; LOD, limit of detection; LR, linear range; MAb, monoclonal antibody; MIF, molecularly imprinted film; MIP, molecularly imprinted polymer; OTA, ochratoxin A; POF, plastic optical fiber; rGO, reduced graphene oxide; SAM, self-assembled monolayer; TNT, 2,4,6-trinitrotoluene

**Table 2:** Examples of SERS-based methods using different recognition elements. The limit of detection (LOD), calculated as the blank response plus three times the standard deviation, is reported.

Analyte	Recognition Element	Assay Format	Immobilization	LOD	Sample Matrix	References
PCB-47	Alkanethiols (C8, C10, C16, C18)	Direct	SAMs of alkanethiols built over AgFON	5 mM	–	[185]
PCB-3 PCB-29 PCB-77	β-CD	Direct	β-CD adsorbed over SiO_2_@Au@AgNPs and immobilized on quartz slides	1 μM	-	[190]
Malathion	Aptamer	Direct	Thiol-modified aptamer on AgNPs@SP	5–100 × 10^−7^ M	Tap water	[191]
Isocarbophos^(a)^ Omethoate^(b)^ Phorate^(c)^ Profenofos^(d)^	Aptamer	Direct	Thiolated aptamer on Ag dendrites	3.4 μM^(a)^ 24 μM^(b)^ 0.4 μM^(c)^ 14 μM^(d)^	Apple juice	[192]
s-propanolol	MIP	Direct	MIP attached to the SERS-active klarite^®^ substrate	7.7 × 10^−4^ M	Urine	[193]
Ciprofloxacin	MIP	Direct	Fe_3_O_4_ NPs@MIP dispersed on a silver solution	10^−9^ M (Water) 10^−7^ M (Serum)	Water Serum	[194]

Abbreviations: β-CD, β-cyclodextrin; AgFON, roughened silver film over nanosphere; AgNPs, silver nanoparticles; AuNPs, gold nanoparticles; C8, n-octane; C10, n-decane; C16, n-hexadecane; C18, n-octadecano; Fe_3_O_4_, iron (II,III) oxide; MIP, molecularly imprinted polymer; PCB-3-chlorobiphenyl; PCB-29, 2,4,5-trichlorobiphenyl; PCB-47, 2,2′,4,4′-tetrachlorobiphenyl; PCB-77, 3,3′,4,4′-tetrachlorobiphenyl; SAM, self assembled monolayer; SiO_2_, silicon dioxide.

**Table 3:** Examples of interferometric-based methods using different recognition elements. The limit of detection (LOD), calculated as the blank response plus three times the standard deviation, is reported. Alternatively, the measured concentration range is shown between brackets.

Analyte	Recognition Element	Assay Format	Immobilization	Sensitivity	Sample Matrix	Interf. Type	References
Domoic acid	PAb	Inhibition	Covalent binding of domoic acid by active ester chemistry	2.0 ng/mL (IC_50_)	Mussel tissue extracts	BLI	[218]
Irgarol 1051	PAb	Binding inhibition	Covalent binding of a derivative onto an APTES functionalized surface	3 ng/L	Sea water	BiMW	[219]
DON	MAb	Competitive	Adsorption of DON-OVA on the sensor chip	128 µg/kg 737 µg/kg	Wheat Wheat dust	BLI	[248]
Testosterone	MAb	Binding inhibition	Covalent binding by active ester chemistry of a derivative onto a DAPEG coated surface	94.4 ng/L	Milk	RIfS	[223]
AFM_1_	Fab′	Competitive	Fab′ binding to a mercaptosilane functionalized surface	5 × 10^–7^ RIU	Milk	aMZI	[207]
Vancomycin	Peptide	Direct	Covalent binding of the peptide to a pSi surface via a carboxy-linker	*K*_D_ = 1.09 × 10^–5^ mol/L	-	RIFTS	[249]
Testosterone	Aptamer	Competitive	Biotinylated aptamer on streptavidin-coated chip	[2.13–136.63 ng/mL]	-	BLI	[234]
Argininamide	Aptamer	Direct	Biotinylated aptamer on avidin-coated chip	5 µM	-	DPI	[236]
Microcystin-LR (MC-LR)	MIP	Direct	Dip coating of the sol–gel matrix	[0.3–1.4 g/L]	Environmental water	FP	[250]
Melamine	MIP	Direct	Spin coating	1251 rad/RIU	-	YI	[208]
Methyl-parathion	AChE	Direct	Covalent attachment to a APTES-GLU-functionalized surface	2.4 × 10^−10^ M (2.1 nM–47 µM)	-	ITFS	[208]

Abbreviations: AChE, acetylcholinesterase; AFM_1_, aflatoxin M_1_; aMZI, asymmetric Mach-Zehnder interferometer; APTES-GLU, 3-aminopropyltriethoxysilane-glutaraldehyde; BiMW, bimodal waveguide interferometer; BLI, biolayer interferometry; DAPEG, diamino-poly(ethylene glycol); DON, deoxynivalenol; DPI, dual polarization interferometer; Fab′, antibody fragments; FP, Fabry-Perot interferometer; IC_50_, half maximal inhibitory concentration; ITFS, interferometric tapered fiber sensor; *K*_D_, equilibrium dissociation constant; mAb, monoclonal antibody; MIP, molecularly imprinted polymer; OVA, ovalbumin; PAb, polyclonal antibody; pSi, porous silicon; RIFTS, reflective interferometric Fourier transform spectroscopy; RIU, refractive index unit; YI, Young’s interferometer.

**Table 4:** Comparative table of different optical label-free biosensors for the detection of ochratoxin A (OTA) as an example of a small molecule.

Method	Recognition Element	Assay Format	Immobilization	Sensitivity	Measurement	Regeneration	Assay Time	Sample	References
SPR	MAb	Direct	GHBs on electropolymerized thionine (PTh) film	LOD 0.01 ng/mL	ESPR with SPR sensor in a flow cell	Up to eight times with glycine-HCl solution (pH 2.8)	<30 min	Milk	[70]
iSPR	MAb	Inhibition	Covalently immobilized toxins on CMD surface	LOD 3 ng/mL	iSPR instrument with nanostructured gold chips	More than 450 times with 10 mM HCl	≈15 min	Beer	[108]
LSPR	Aptamer	Direct	Aptamer with 5′ thiol on Au surface	<1 nM (=0.4 ng/mL)	Microplate reader with 96-well plate	Seven times with 10% methanol at +70 °C	≈15 min	Corn	[118]
OWLS	MAb	Direct and competitive	Covalently immobilized MAb (direct) or OTA-BSA (competitive)	DR 5–10 ng/mL for direct, 0.5–10 ng/mL for competitive	Integrated OWLS sensor chips	n.d.	<10 min	Barley and wheat	[41]
PW (PI principle)	MAb	Direct	Antibody via protein A	DR 0.01–100 ng/mL	PW structures on silicon wafers, CCD array and a polarizer	n.d.	n.d.	–	[217]
SERS	Aptamer	Direct	Aptamer with 5′ thiol on Au surface	DR 0.05–4 μM	SERS platform with microfluidic channel	n.d.	n.d.	–	[251]

Abbreviations: BSA; bovine serum albumin, CCD; charge-coupled device (CCD), CMD; 3-dimensional carboxymethylated dextran, DON; deoxynivalenol, DR; detection range, ESPR; electrochemical surface plasmon resonance, GHB; gold hollow balls, LOD; limit of detection, MAb; monoclonal antibody, iSPR; imaging surface plasmon resonance, OWLS; optical waveguide lightmode spectroscopy, PI; polarization interferometry, PW; planar waveguide.
